# An early geikiid dicynodont from the *Tropidostoma* Assemblage Zone (late Permian) of South Africa

**DOI:** 10.7717/peerj.2913

**Published:** 2017-01-31

**Authors:** Christian F. Kammerer, Roger M.H. Smith

**Affiliations:** 1 Museum für Naturkunde, Leibniz-Institut für Evolutions- und Biodiversitätsforschung, Berlin, Germany; 2 Evolutionary Studies Institute, University of the Witwatersrand, Johannesburg, South Africa; 3 Iziko Museums of South Africa, Cape Town, South Africa

**Keywords:** Synapsida, Therapsida, Dicynodontia, Permian, Karoo Basin, South Africa, Ghost lineage, Phylogeny

## Abstract

Based on specimens previously identified as *Tropidostoma*, a new taxon of dicynodont (*Bulbasaurus phylloxyron* gen. et sp. nov.) from the Karoo Basin of South Africa is described. *Bulbasaurus* is a medium-sized dicynodont (maximum dorsal skull length 16.0 cm) restricted to the *Tropidostoma* Assemblage Zone (early Lopingian) of the Beaufort Group. *Bulbasaurus* can be distinguished from *Tropidostoma* by an array of characters including the presence of a tall, sharp premaxillary ridge, large, rugose, nearly-confluent nasal bosses, a nasofrontal ridge, massive tusks, robust pterygoids, prominently twisted subtemporal bar, and absence of a distinct postfrontal. Inclusion of *Bulbasaurus* in a phylogenetic analysis of anomodont therapsids recovers it as a member of Geikiidae, a clade of otherwise later Permian dicynodonts such as *Aulacephalodon* and *Pelanomodon*. *Bulbasaurus* exhibits many of the characters typical of adult *Aulacephalodon*, but at substantially smaller skull size (these characters are absent in comparably-sized *Aulacephalodon* juveniles), suggesting that the evolution of typical geikiid morphology preceded gigantism in the clade. *Bulbasaurus* is the earliest known geikiid and the only member of the group known from the *Tropidostoma* Assemblage Zone; discovery of this taxon shortens a perplexing ghost lineage and indicates that abundant clades from the later Permian of South Africa (e.g., Geikiidae, Dicynodontoidea) may have originated as rare components of earlier Karoo assemblage zones.

## Introduction

Several major turnovers in tetrapod faunal composition occurred in the Karoo Basin of South Africa during the span of time recorded in the Permo-Triassic Beaufort Group (Rubidge, 1995). The best-known and most intensely-studied of these turnovers corresponds to the end-Permian mass extinction, which wiped out the majority of species and several major clades of tetrapods (Smith & Ward, 2001; Retallack, Smith & Ward, 2003; Ward et al., 2005; Botha-Brink & Angielczyk, 2010; Irmis & Whiteside, 2012; Fröbisch, 2013; Smith & Botha-Brink, 2014). Recently, however, increased research attention has also been given to the turnover between middle (Guadalupian) and late (Lopingian) Permian Karoo faunas. Studies of this transition have primarily focused on the role of extinction in driving faunal change, and the possible relationship between Karoo tetrapod turnover and global marine turnover as part of a hypothesized mid-Permian mass extinction (Rubidge et al., 2013; Rubidge et al., 2016; Day et al., 2015a; Day et al., 2015b; Réy et al., 2016). However, the primary victims of mid-Permian extinction among Karoo tetrapods were the dinocephalian therapsids, a group that, although ecologically diverse and species-rich (Boonstra, 1969; Kammerer, 2011), represent a relatively small component of middle Permian faunas in terms of specimen abundance (Smith, Rubidge & van der Walt, 2012). The most abundant middle Permian Karoo therapsids were, just as in the late Permian and earliest Triassic, the anomodonts, and specifically their subclade Dicynodontia. Dicynodonts suffered minimal extinction-related turnover between the middle and late Permian. Indeed, the most abundant middle Permian dicynodont (the pylaecephalid *Diictodon feliceps*) only becomes more abundant in late Permian strata (Smith, Rubidge & van der Walt, 2012) (before finally trailing off and going extinct in the terminal Permian *Daptocephalus* Assemblage Zone; Viglietti et al., 2016). The next-most-abundant small-bodied Permian dicynodonts (*Pristerodon* and *Emydops*) also first appear in the middle Permian (Angielczyk, Fröbisch & Smith, 2005; Kammerer, Angielczyk & Fröbisch, 2011) and survive the mid-Permian extinction seemingly unscathed. Among dicynodont taxa whose ranges do not extend into the late Permian, most are components of the earliest Karoo faunal assemblages, none of whose members even make it to the end of the middle Permian (e.g., *Eodicynodon*, *Lanthanostegus*, *Colobodectes*) (Modesto, Rubidge & Welman, 2002; Angielczyk & Rubidge, 2009). Furthermore, the few dicynodont taxa that are apparent victims of the mid-Permian extinction (*Brachyprosopus broomi* and *Robertia broomiana*) have close relatives (at the ‘family level’) that thrive in the late Permian (Angielczyk & Rubidge, 2013; Cox & Angielczyk, 2014; Angielczyk et al., 2016).

Even though dicynodonts show robust survivorship between the middle and late Permian, there are nonetheless major differences between middle and late Permian Karoo dicynodont faunas. However, these differences are not driven by extinction within the clade, but rather by origination, and specifically the appearance of large-bodied taxa. All middle Permian dicynodonts are relatively small (skull length <20 cm) animals; the niche of large herbivore in the middle Permian Karoo was restricted to dinocephalians and pareiasaurs (Boonstra, 1969). In the late Permian, however, dicynodonts are overwhelmingly the most abundant large herbivores (Kammerer, Angielczyk & Fröbisch, 2011; Smith, Rubidge & van der Walt, 2012). Although this may have been driven in part by the opening up of ecospace resulting from the extinction of herbivorous dinocephalians, there is a substantial lag between dinocephalian extinction and large dicynodont evolution. Dinocephalians go extinct at the end of the *Tapinocephalus* Assemblage Zone (AZ) (Day et al., 2015a). Only one large-bodied dicynodont (the aberrant *Endothiodon bathystoma*; Cox & Angielczyk, 2014) appears immediately in the wake of dinocephalian extinction, in the overlying *Pristerognathus* AZ, and few large-bodied dicynodonts are present in the subsequent *Tropidostoma* AZ. The majority of large-bodied dicynodont taxa do not appear until the later *Cistecephalus* and *Daptocephalus* AZs, roughly five million years afterwards (Smith, Rubidge & van der Walt, 2012; Rubidge et al., 2013; Viglietti et al., 2016).

With the notable exception of the aforementioned *Endothiodon*, almost all large (>30 cm skull length) dicynodont herbivores belong to the clade Bidentalia. Bidentalians are characterized by a reduction in dentition relative to earlier dicynodonts (in almost all taxa only the maxillary tusks are present, if teeth are present at all) and include a number of abundant and well-known Karoo taxa such as *Dicynodon*, *Daptocephalus*, *Oudenodon*, and *Aulacephalodon* (Kammerer & Angielczyk, 2009). In South Africa, this clade first appears in the *Tropidostoma* AZ, in the form of the eponymous *Tropidostoma dubium*, a medium-sized oudenodontid (adult skull length: ∼20 cm; Botha & Angielczyk, 2007). The *Tropidostoma* AZ is a crucial turning-point for Karoo therapsid faunas in general: cynodonts, which would go on to become the most successful Mesozoic therapsids, first appear in this assemblage zone (Botha, Abdala & Smith, 2007; Kammerer, 2016), and eutherocephalians and gorgonopsians, the dominant predators of the later Permian, show a marked increase in abundance (Smith, Rubidge & van der Walt, 2012; Kammerer et al., 2015). The primary bidentalian component of the *Tropidostoma* AZ is *Tropidostoma* itself, although rare records of *Oudenodon* and *Rhachiocephalus* also appear in the upper part of this assemblage zone (Botha & Angielczyk, 2007; Smith, Rubidge & van der Walt, 2012). Perplexing in their absence, however, are any members of the clades Dicynodontoidea or Geikiidae. Dicynodontoids represent one of the two major bidentalian subclades (the other being Cryptodontia, which includes the aforementioned *Tropidostoma*, *Oudenodon*, *Rhachiocephalus*, and allied taxa) and are very conspicuous members of later assemblage zones (including the nominal genera for the *Daptocephalus* and *Lystrosaurus* AZs). Geikiids are a cryptodont group, and include the abundant and highly autapomorphic large-bodied taxon *Aulacephalodon bainii*, which first appears in the *Cistecephalus* AZ (Tollman, Grine & Hahn, 1980; Kammerer, Angielczyk & Fröbisch, 2011; Kammerer, Angielczyk & Fröbisch, 2015a).

Based on the presence of their sister-clades in the *Tropidostoma* AZ, dicynodontoids and geikiids must have diverged by this time. There are three main explanations for the absence of these taxa in the *Tropidostoma* AZ of South Africa. One is that these taxa were present but rare components of the fauna, and sampling effort has not been sufficient to recover their remains. However, the *Tropidostoma* AZ is a very well-sampled assemblage zone (Smith, Rubidge & van der Walt, 2012), and has already yielded specimens of even extremely rare therapsid taxa such as burnetiamorphs (Sidor, Hopson & Keyser, 2004; Sidor & Smith, 2007). A second explanation is that the missing taxa did not originate in South Africa, and only dispersed to the Karoo Basin later in their evolution. This is an important possibility to consider, given how little we know about late Permian tetrapod faunas globally, and the fact that bidentalians were already also present in Russia in probable *Tropidostoma* AZ-equivalent strata (Kurkin, 2000; Angielczyk & Kurkin, 2003). There is a third possibility, however—that these taxa were present in the *Tropidostoma* AZ and their fossils have been collected, but not recognized as such. South African dicynodont alpha taxonomy has only recently been stabilized after roughly a century in the wilderness (see Kammerer, Angielczyk & Fröbisch (2011) for a review). In the absence of meaningful species diagnoses in the wake of Broomian hyper-splitting in the early 20th century, researchers working on Karoo biostratigraphy tended to refer newly-collected dicynodont specimens to a few, broadly-understood dicynodont genera (Kitching, 1977; Cluver & Hotton, 1981). Now that the majority of nominal taxa have been resolved into well-diagnosed morphospecies, these broad earlier specimen referrals can be reevaluated, and in some cases have proven to be over-lumped, creating heterogeneous arrays of unrelated taxa (Kammerer, Angielczyk & Fröbisch, 2011; Kammerer, Angielczyk & Fröbisch, 2015b). This is particularly problematic when dealing with basal members of highly apomorphic clades, i.e., taxa that do not yet show the full suite of synapomorphies traditionally used to characterize those clades. Specimens of such species may instead end up referred to coeval, generalized dicynodont taxa, obscuring their importance as early representatives of clades that would later become major components of their faunas (e.g., the earliest lystrosaurid *Euptychognathus*, originally referred to ‘*Dicynodon*’; Kammerer, Angielczyk & Fröbisch, 2011). Here, we present another such case, recognizing the first geikiid dicynodont from the *Tropidostoma* AZ on the basis of specimens previously identified as *Tropidostoma* itself.

## Materials and Methods

All specimens mentioned in the text were examined personally by the lead author. For information on the methodology of the phylogenetic analysis, refer to the Discussion. The electronic version of this article in Portable Document Format (PDF) will represent a published work according to the International Commission on Zoological Nomenclature (ICZN), and hence the new names contained in the electronic version are effectively published under that Code from the electronic edition alone. This published work and the nomenclatural acts it contains have been registered in ZooBank, the online registration system for the ICZN. The ZooBank LSIDs (Life Science Identifiers) can be resolved and the associated information viewed through any standard web browser by appending the LSID to the prefix http://zoobank.org/. The LSID for this publication is: urn:lsid:zoobank.org:pub:EB1EE1F8-C512-4272-BFA3-C8938E0806E5. The online version of this work is archived and available from the following digital repositories: PeerJ, PubMed Central and CLOCKSS.

The holotype specimen of the new taxon (SAM-PK-K11235) was collected by one of the authors (RMHS) in *Tropidostoma* AZ exposures of the locality Vredelus (Fraserburg District, Western Cape Province). The *Tropidostoma* AZ strata in the southern Karoo Basin mainly coincide with the argillaceous Hoedemaker Member (Teekloof Formation), which represents the distal portion of a large distributary fluvial system. Three dimensional outcrops of exhumed paleomeanderbelts (the Reiersvlei Sandstone of Smith (1987)) in the vicinity of the type locality of the new dicynodont show that the former rivers had channel widths of up to 350 m, and point bar diameters of ∼3 km.

**Figure 1:**
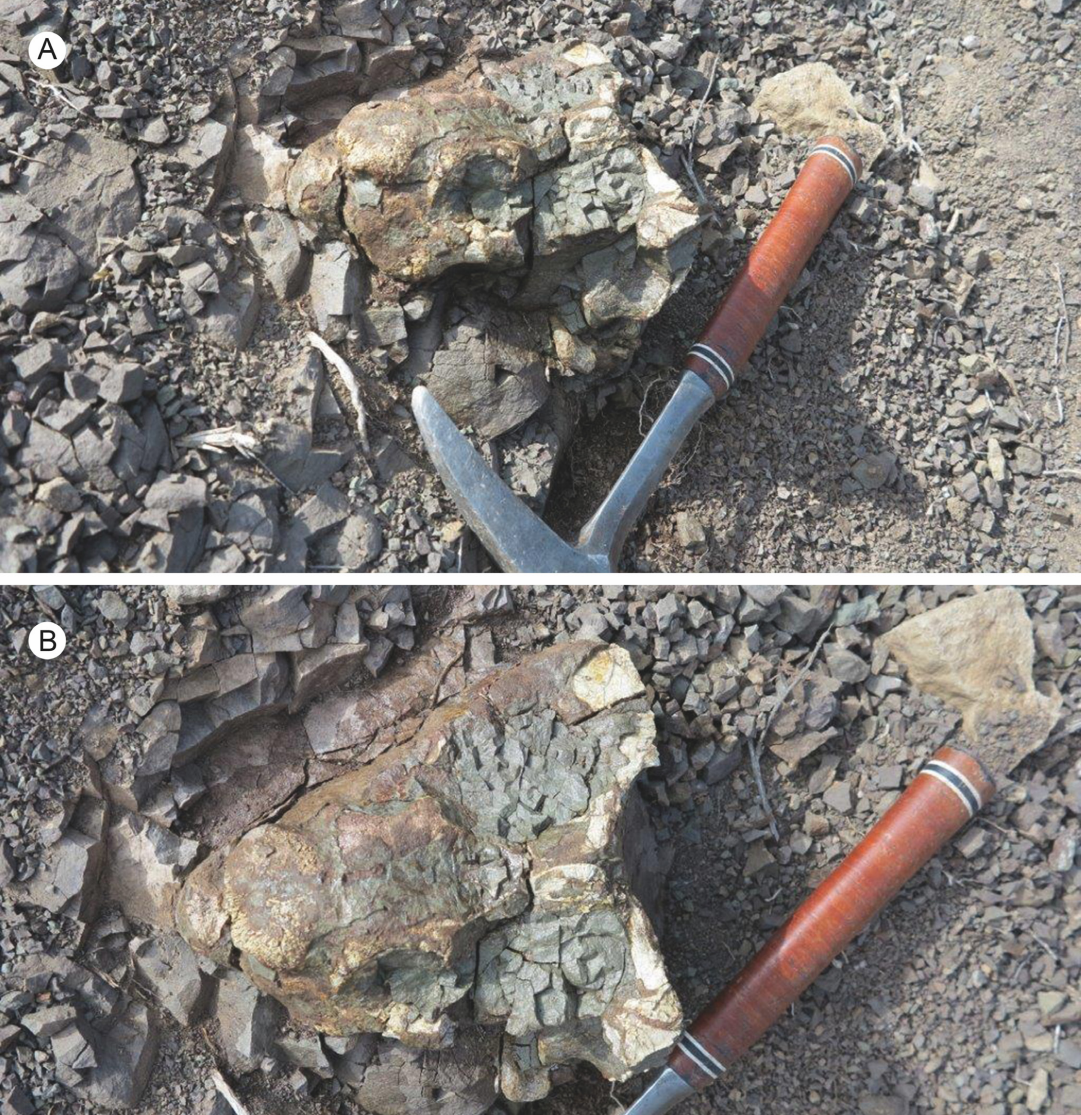
SAM-PK-K11235, holotype of *Bulbasaurus phylloxyron* gen. et sp. nov., *in situ* at the type locality of Driekoppe, Vredelus, Fraserburg, Western Cape Province, South Africa. (A) Specimen in left semi-lateral view; (B) specimen in dorsal view.

SAM-PK-K11235 was preserved dorsal-side-up in a 1.5 m thick bed of structureless grey siltstone (Fig. 1) with scattered small micrite nodules immediately overlying a 1 m-thick fine-grained ripple cross-laminated sandstone with mudrock rip-up clasts. The massive siltstone is interpreted as having been deposited on the proximal floodplain alongside a large northeasterly-flowing meandering river on one of the low angled fluvial fans that emanated from the Gondwanide mountains. The linear channel-fill sandstone beneath represents a minor floodplain distributary.

The holotype skull was found *in situ* with no skeletal elements in association and was not perimineralized with calcareous nodular material. This ‘head-only’ taphonomic style is typical of therapsid occurrences in proximal floodplain deposits of the Hoedemaker Member (Smith, 1993) and is attributed to extended periods of non-deposition, allowing for sub-aerial disarticulation of skeletons, then short transportation and rapid burial by silt-laden overbank floods.

SAM-PK-K11235 was mechanically prepared by Mr. Sibusiso Mtungata in the Karoo palaeontology laboratory, Iziko Museums of South Africa (Cape Town). All other specimens referred to the new dicynodont taxon were also mechanically prepared, either at Iziko or the Council for Geoscience (Pretoria).

## Systematic Paleontology

**Table:** 

**Synapsida** Osborn, 1903
**Therapsida** Broom, 1905
**Anomodontia** Owen, 1860
**Dicynodontia** Owen, 1860
**Cryptodontia** Owen, 1860
**Geikiidae** Nopcsa, 1923

*Type genus*: *Geikia* Newton, 1893.

*Included genera*: *Aulacephalodon* Seeley, 1898; *Bulbasaurus* gen. nov.; *Pelanomodon* Broom, 1938.

***Bulbasaurus*** **gen. nov.**

*LSID*: urn:lsid:zoobank.org:act:F21D10AF-277A-44D4-B374-BE89AF2B7541

*Type species*: *Bulbasaurus phylloxyron* sp. nov.

*Etymology*: From the Latin *bulbus* (a bulb), referring to the shape of the nasal boss, and the latinized Greek *saurus* (a lizard), a common suffix for fossil amniotes of ‘reptilian’ grade.

*Diagnosis*: As for the type and only species.

***Bulbasaurus phylloxyron*** **sp. nov.**

Figs. 1–16

*LSID*: urn:lsid:zoobank.org:act:C80D31CA-86B1-43C4-9EA9-CED4A5852262

**Figure 2:**
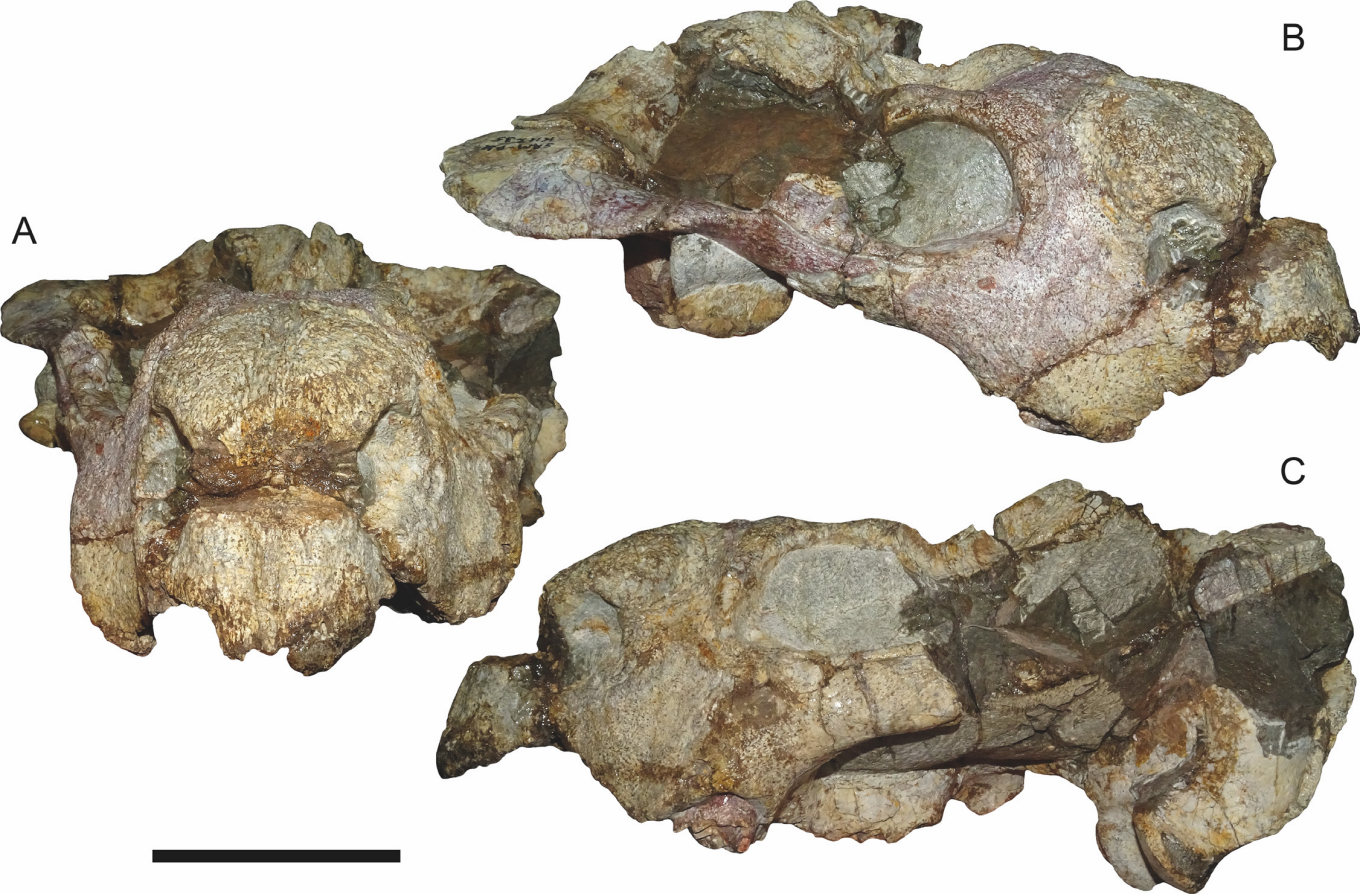
SAM-PK-K11235, holotype of *Bulbasaurus phylloxyron* gen. et sp. nov., in (A) anterior, (B) right semi-lateral, and (C) left lateral views. Scale bar equals 5 cm.

**Figure 3:**
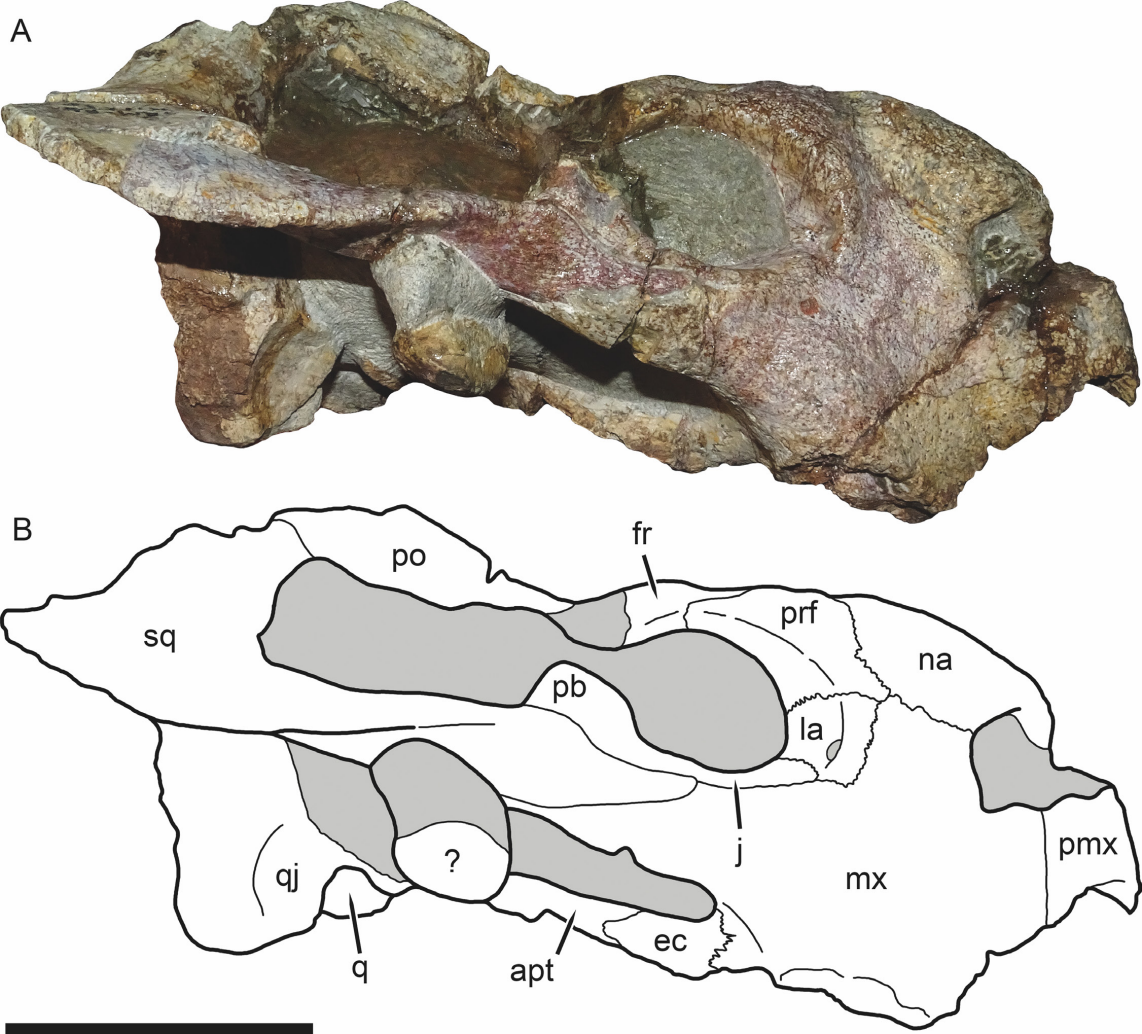
SAM-PK-K11235, holotype of *Bulbasaurus phylloxyron* gen. et sp. nov., in right lateral view. (A) photograph and (B) interpretive drawing. Abbreviations: ?, unknown bone; apt, anterior pterygoid ramus; ec, ectopterygoid; fr, frontal; la, lacrimal; mx, maxilla; na, nasal; pb, base of postorbital bar; pmx, premaxilla; po, postorbital; prf, prefrontal; q, quadrate; qj, quadratojugal; sq, squamosal. Gray indicates matrix. Scale bar equals 5 cm.

**Figure 4:**
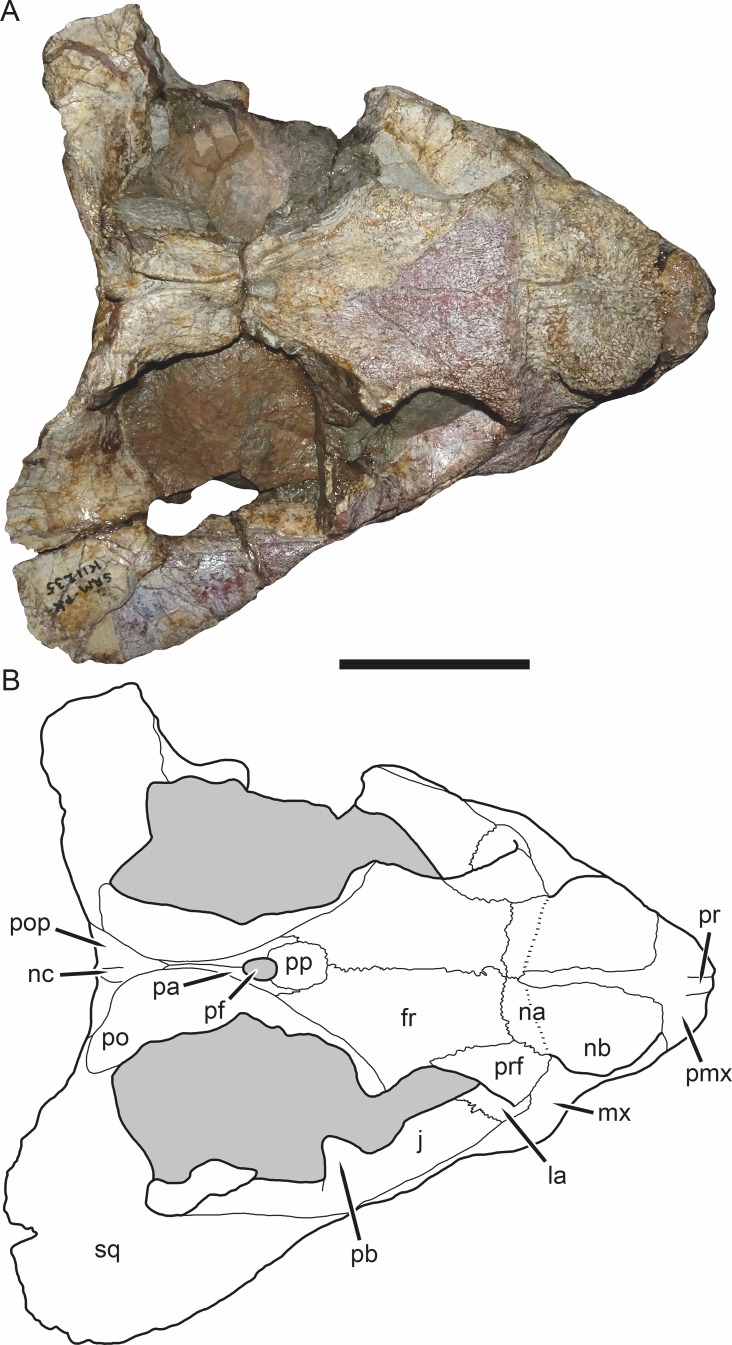
SAM-PK-K11235, holotype of *Bulbasaurus phylloxyron* gen. et sp. nov., in dorsal view. (A) photograph and (B) interpretive drawing. Abbreviations: fr, frontal; la, lacrimal; j, jugal; na, nasal; nb, nasal boss; nc, nuchal crest; pa, parietal; pb, base of postorbital bar; pf, pineal foramen; pmx, premaxilla; po, postorbital; pop, postparietal; pp, preparietal; pr, premaxillary ridge; prf, prefrontal; sq, squamosal. Gray indicates matrix. Scale bar equals 5 cm.

**Figure 5:**
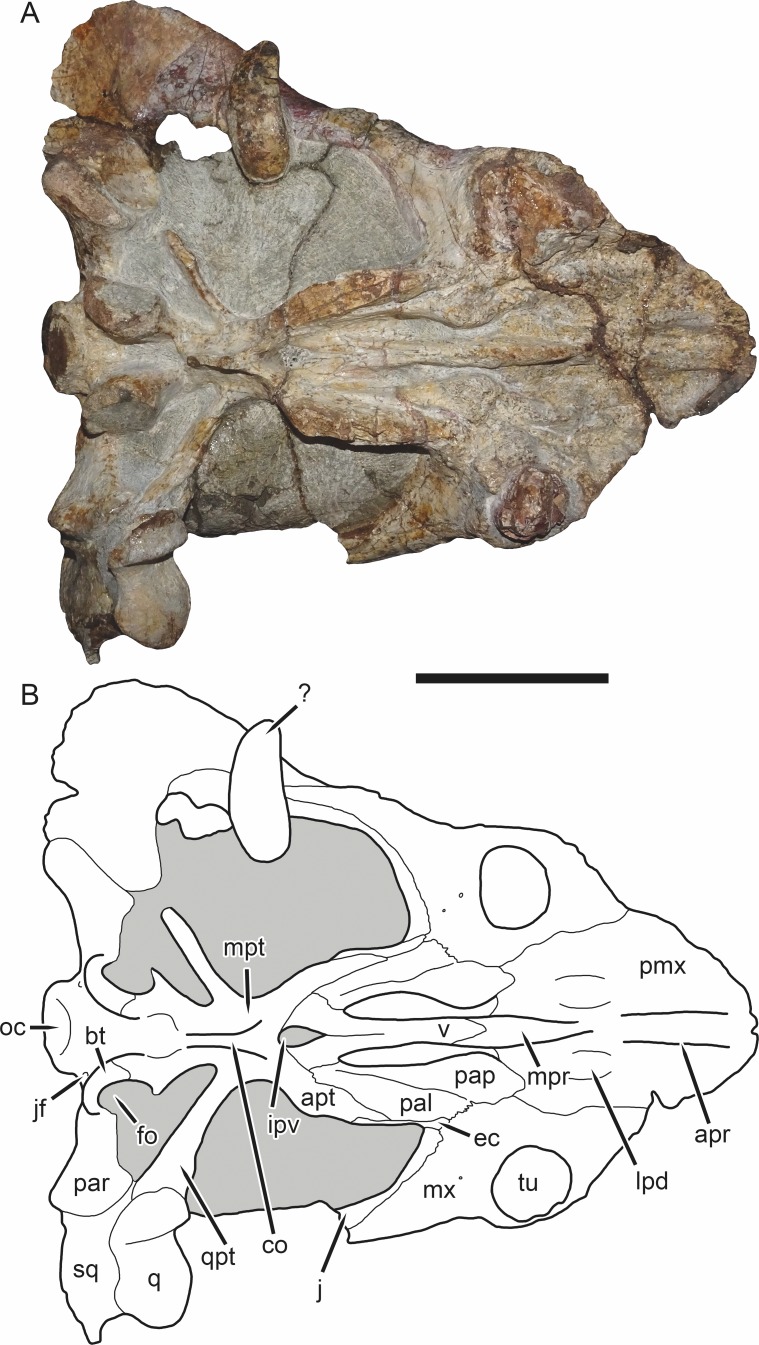
SAM-PK-K11235, holotype of *Bulbasaurus phylloxyron* gen. et sp. nov., in ventral view. (A) photograph and (B) interpretive drawing. Abbreviations: ?, unknown bone; apr, anterior palatal ridge; apt, anterior pterygoid ramus; bt, basal tuber; co, crista oesophagea; ec, ectopterygoid; fo, fenestra ovalis; ipv, interpterygoid vacuity; j, jugal; jf, jugular foramen; lpd, lateral premaxillary depression; mpr, median palatal ridge; mpt, median pterygoid plate; mx, maxilla; oc, occipital condyle; pal, palatine; pap, palatine pad; par, paroccipital process; pmx, premaxilla; q, quadrate; qpt, quadrate ramus of pterygoid; sq, squamosal; tu, tusk; v, vomer. Gray indicates matrix. Scale bar equals 5 cm.

**Figure 6:**
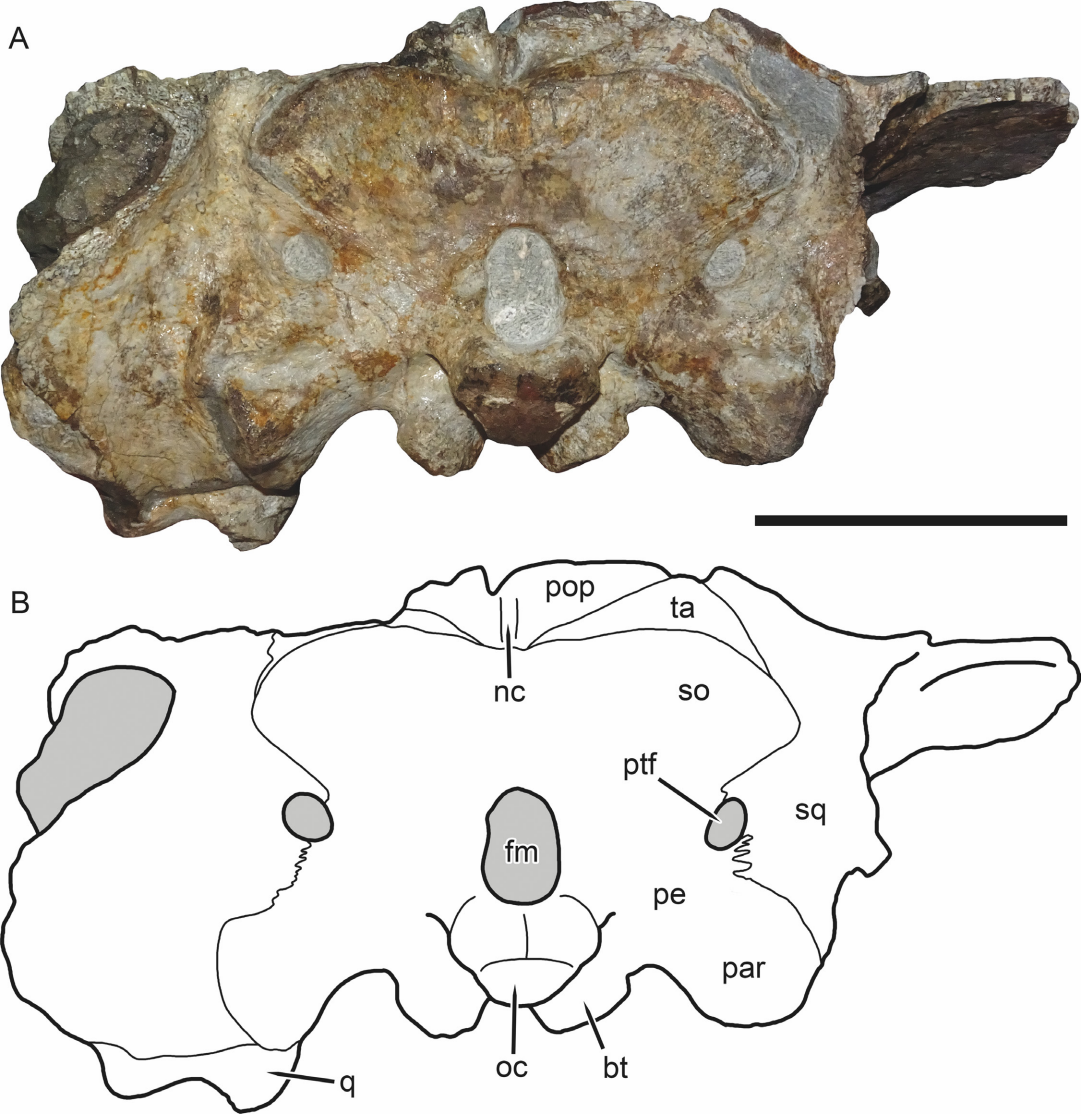
SAM-PK-K11235, holotype of *Bulbasaurus phylloxyron* gen. et sp. nov., in occipital view. (A) photograph and (B) interpretive drawing. Abbreviations: bt, basal tuber; fm, foramen magnum; nc, nuchal crest; oc, occipital condyle; par, paroccipital process; pe, periotic; pop, postparietal; ptf, post-temporal fenestra; q, quadrate; so, supraoccipital (exact boundaries uncertain, fused with periotic); sq, squamosal; ta, tabular. Gray indicates matrix. Scale bar equals 5 cm.

*Holotype*: SAM-PK-K11235, a partial skull missing the left subtemporal bar and both postorbital bars (Figs. 1–6).

*Type locality and horizon*: Driekoppe, Vredelus, Fraserburg, Western Cape Province, South Africa. Hoedemaker Member of the Teekloof Formation, ∼50 m above top of Reiersvlei Sandstone at a height of 1,445 m, *Tropidostoma* Assemblage Zone (Lopingian).

**Figure 7:**
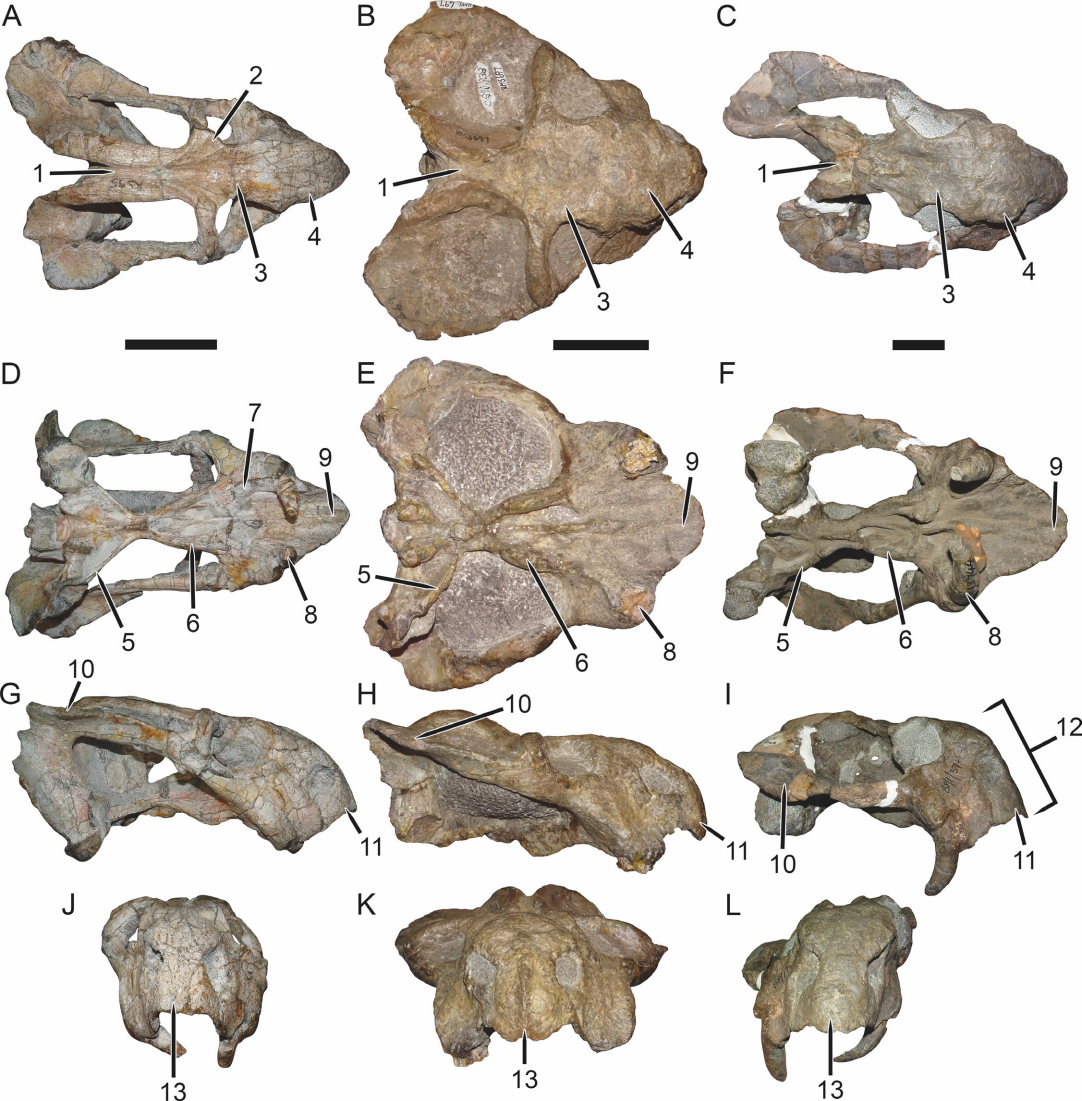
Comparisons of *Bulbasaurus* with *Tropidostoma* and *Aulacephalodon*. Specimens: *Tropidostoma dubium* (SAM-PK-K11238) in (A) dorsal, (D) ventral, (G) right lateral, and (J) anterior views. *Bulbasaurus phylloxyron* (CGP/1/938) in (B) dorsal, (E) ventral, (H) right lateral, and (K) anterior views. *Aulacephalodon bainii* (BP/1/3744) in (C) dorsal, (F) ventral, (I) right lateral, and (L) anterior views. Specimen of *Bulbasaurus* largely undistorted; specimens of *Tropidostoma* and *Aulacephalodon* have suffered some lateral compression. Scale bars equal 5 cm; all figures of an individual specimen to scale with one another. Characters: 1, intertemporal exposure of parietal: elongate, narrow channel in *Tropidostoma*, ‘pinched’ but anteriorly broad in *Bulbasaurus*, and broad throughout but transversely expanded posteriorly in *Aulacephalodon*. 2, postfrontal: large and triangular in *Tropidostoma*, absent in *Bulbasaurus* and large specimens of *Aulacephalodon*. 3, interorbital width: narrow in *Tropidostoma*, relatively broad in *Bulbasaurus* and *Aulacephalodon*. 4, nasal bosses: discrete and widely-separated, transversely narrow, ovoid, smooth bosses in *Tropidostoma*, large, rugose bosses covering almost all of nasal surface and nearly meeting on midline in *Bulbasaurus*, and large, rugose, but discrete and widely-separated bosses in *Aulacephalodon*. 5, quadrate ramus of pterygoid: relatively thin in *Tropidostoma*, relatively thick in *Bulbasaurus* and *Aulacephalodon*. 6, anterior ramus of pterygoid: relatively thin in *Tropidostoma*, relatively thick in *Bulbasaurus* and *Aulacephalodon*. 7, postcanine teeth: present in *Tropidostoma*, absent in *Bulbasaurus* and *Aulacephalodon*. 8, maxillary tusk: relatively small in *Tropidostoma*, massive in *Bulbasaurus*, also massive in *Aulacephalodon* but only at large skull size—specimen figured here is at minimal size for large tusks in the taxon; smaller individuals have thin or just-erupting tusks. 9, depression between anterior palatal ridges: deeper than depressions lateral to ridges in *Tropidostoma*, of equal depth as lateral depressions in *Bulbasaurus* and *Aulacephalodon*. 10, subtemporal bar: with only a slight deflection of the dorsal edge in *Tropidostoma*, ‘twisted’ in *Bulbasaurus*, such that the medial surface of the bar becomes the dorsal surface posteriorly, and strongly ‘twisted’ in *Aulacephalodon*, such that the medial surface of the bar becomes the lateral surface posteriorly. 11, ‘beak’: with only weak curvature in *Tropidostoma* and *Aulacephalodon*, only extreme anterior edge with hooked tip, versus strongly ventrally-curved in *Bulbasaurus*, with most of premaxillary ventral margin making up hooked tip. 12, snout depth: very deep in *Aulacephalodon*, comparatively shallow in *Tropidostoma* and *Bulbasaurus*. 13, anterior premaxillary ridge: absent or weakly-diffuse in *Tropidostoma*, very discrete and well-developed in *Bulbasaurus*, and absent in smaller individuals of *Aulacephalodon* such as the one figured here (and even in the largest adults, the premaxillary ridge of *Aulacephalodon* is proportionally lower and more diffuse than that of *Bulbasaurus*).

**Figure 8:**
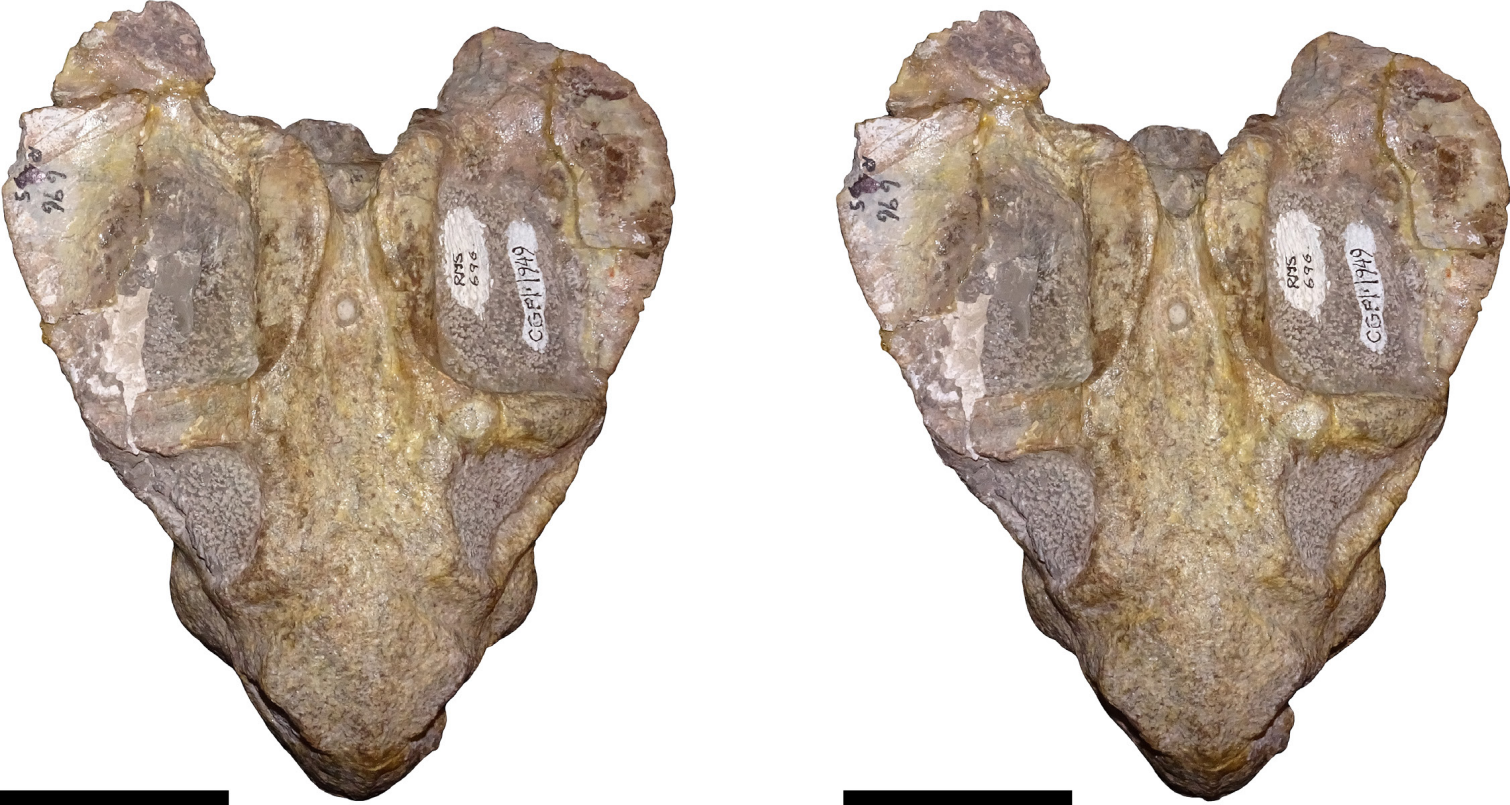
Stereopair of CGP/1/949, referred specimen of *Bulbasaurus phylloxyron* gen. et sp. nov., in dorsal view. Scale bars equal 5 cm.

**Figure 9:**
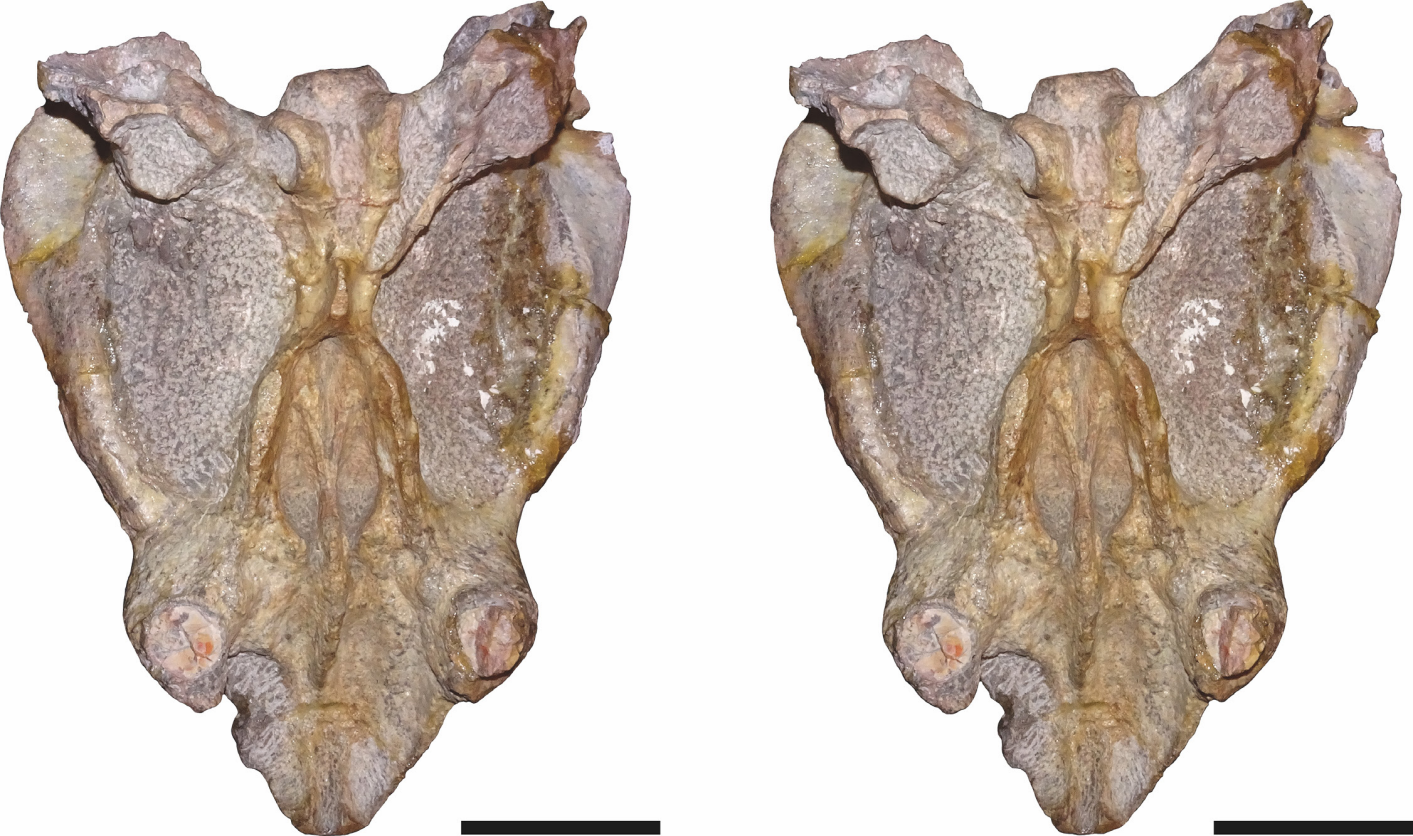
Stereopair of CGP/1/949, referred specimen of *Bulbasaurus phylloxyron* gen. et sp. nov., in ventral view. Scale bars equal 5 cm.

**Figure 10:**
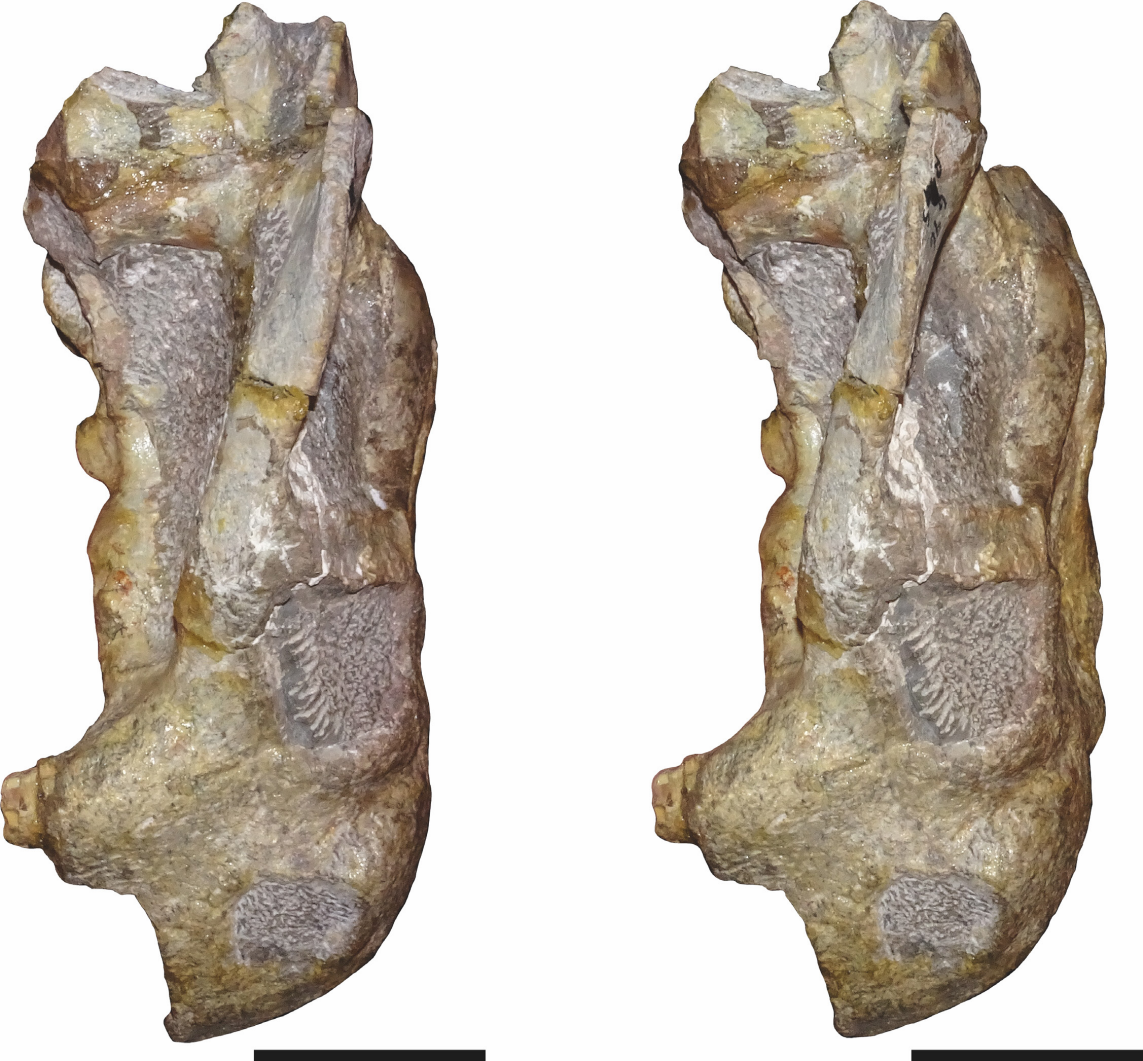
Stereopair of CGP/1/949, referred specimen of *Bulbasaurus phylloxyron* gen. et sp. nov., in right lateral view. Scale bars equal 5 cm.

**Figure 11:**
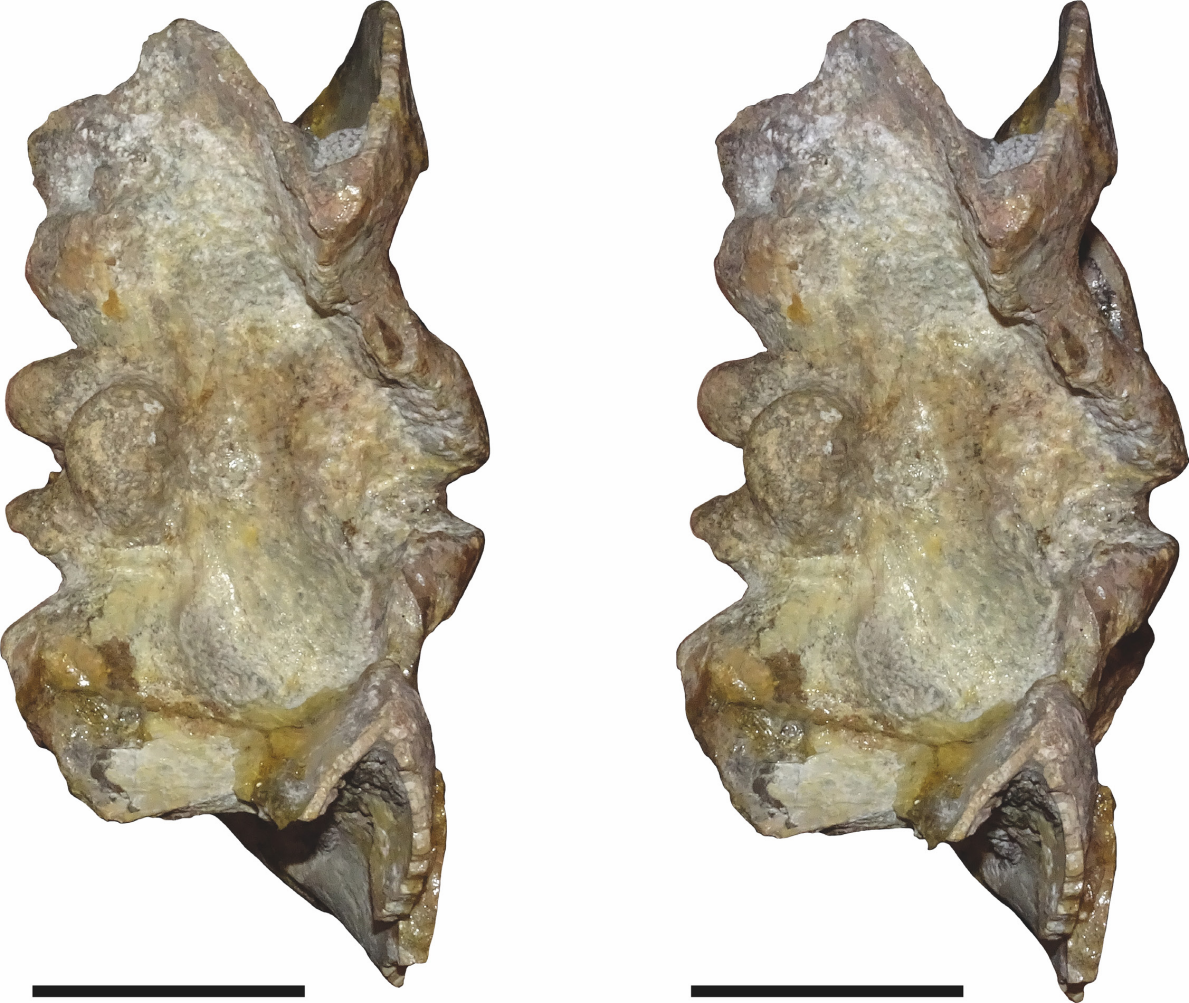
Stereopair of CGP/1/949, referred specimen of *Bulbasaurus phylloxyron* gen. et sp. nov., in occipital view. Scale bars equal 5 cm.

**Figure 12:**
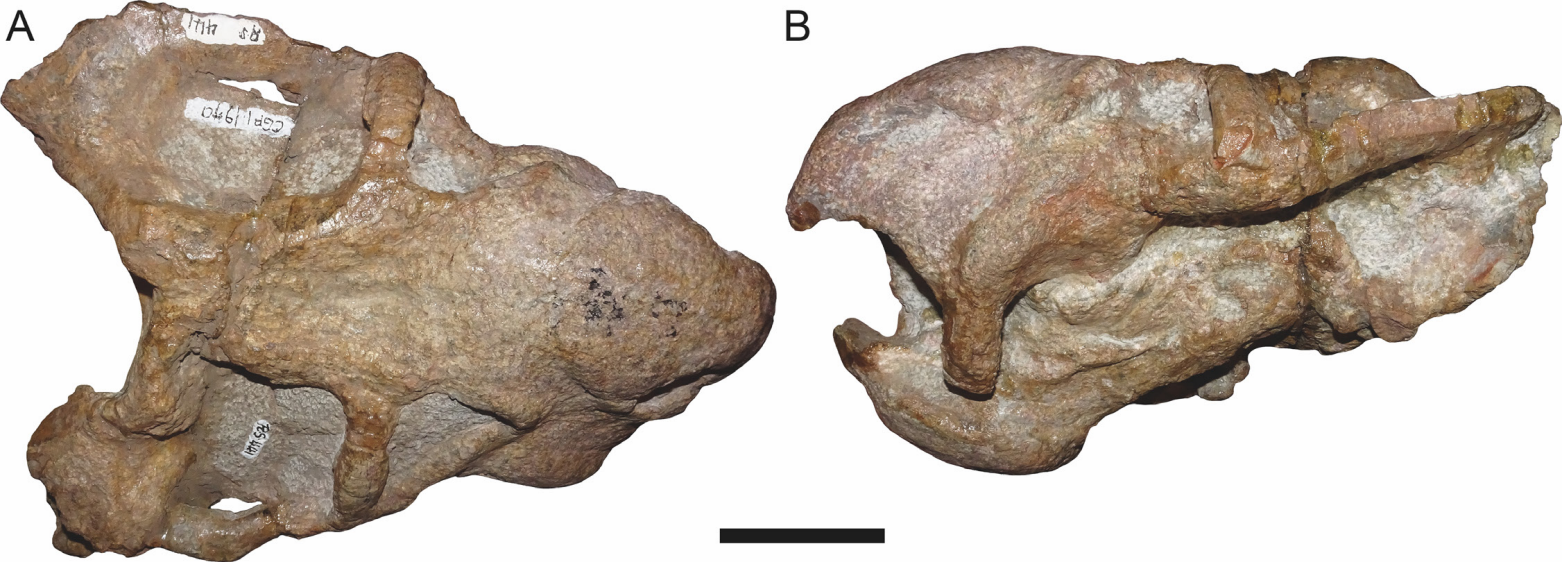
CGP/1/970, referred specimen of *Bulbasaurus phylloxyron* gen. et sp. nov., in (A) dorsal and (B) left lateral views. Postcranial elements edited out to highlight cranial morphology; see Figs. 13 and 14 for postcranium. Scale bar equals 5 cm.

**Figure 13:**
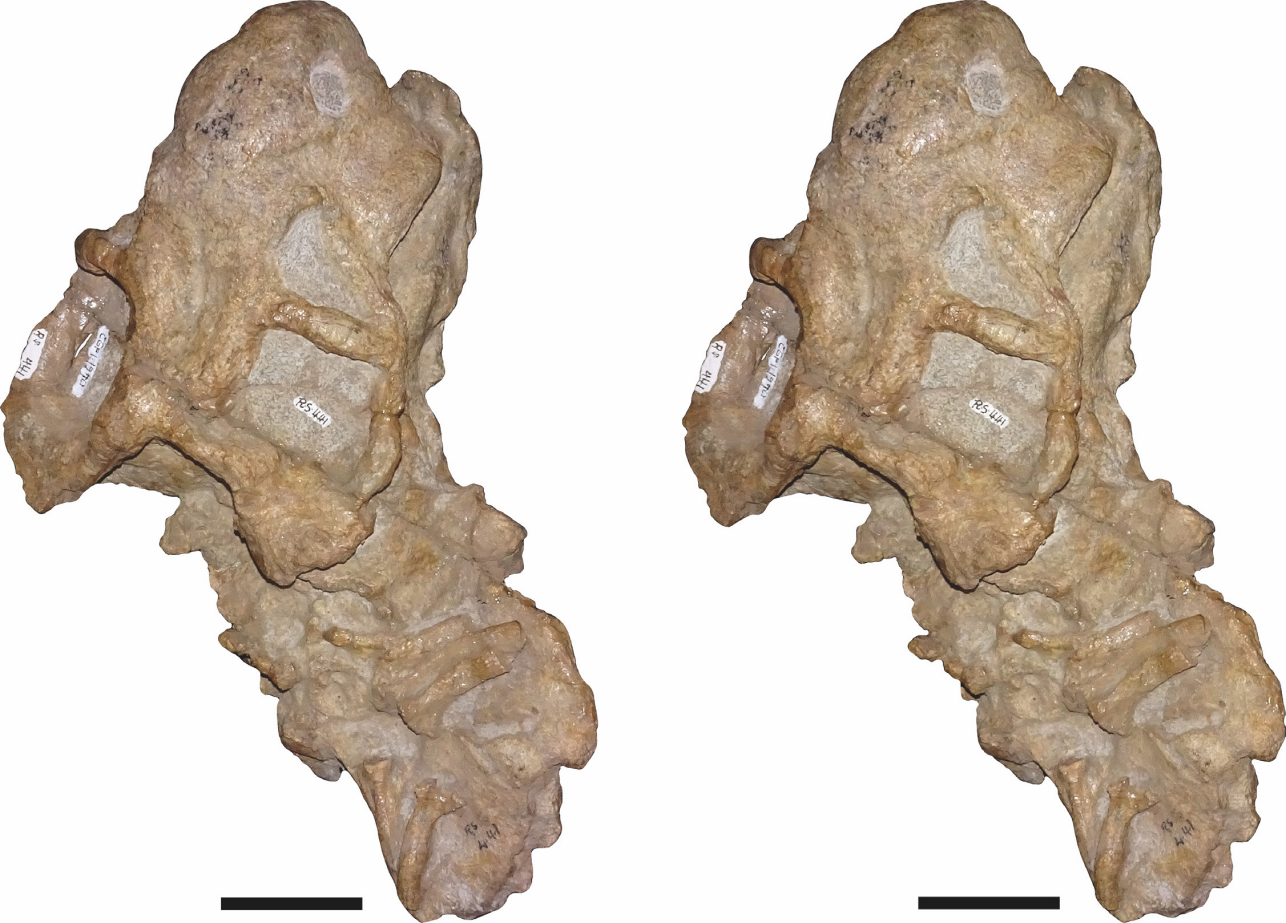
Stereopair of CGP/1/970, referred specimen of *Bulbasaurus phylloxyron* gen. et sp. nov., in semi-dorsal view. Scale bars equal 5 cm.

**Figure 14:**
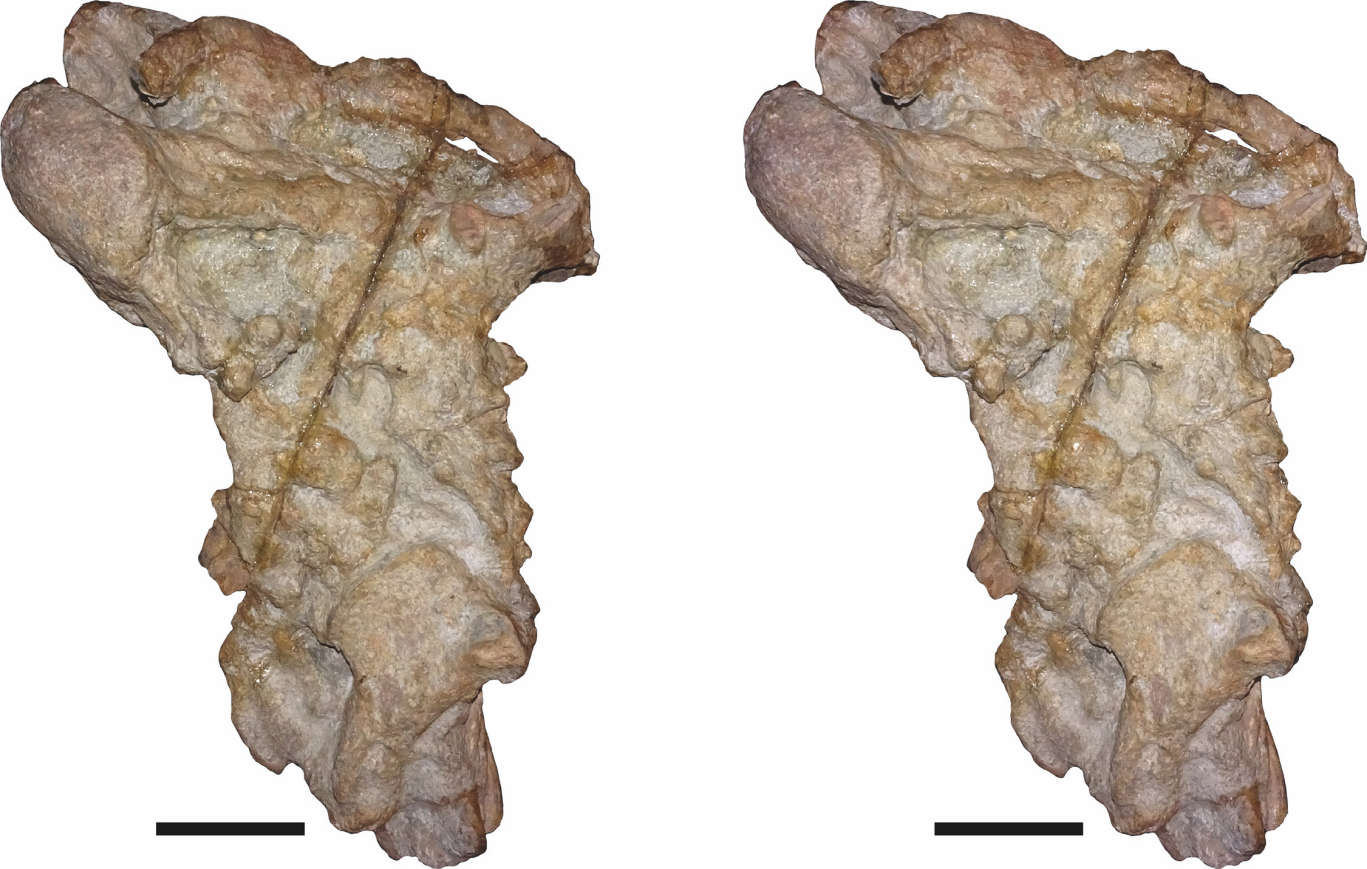
Stereopair of CGP/1/970, referred specimen of *Bulbasaurus phylloxyron* gen. et sp. nov., in ventral view. Scale bars equal 5 cm.

**Figure 15:**
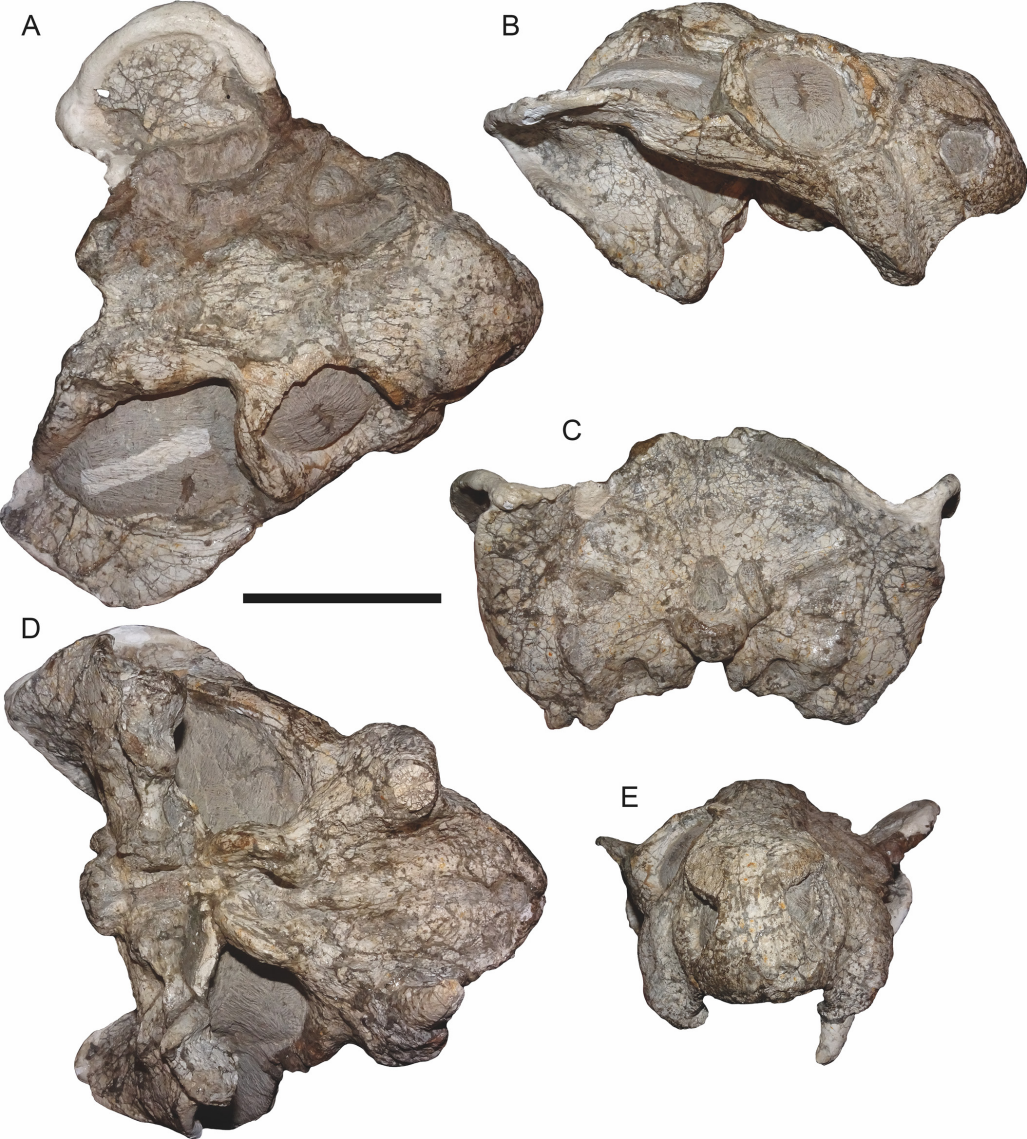
CGP/1/2263, referred specimen of *Bulbasaurus phylloxyron* gen. et sp. nov., in (A) dorsal, (B) right lateral, (C) occipital, (D) ventral, and (E) anterior views. Scale bar equals 5 cm.

**Figure 16:**
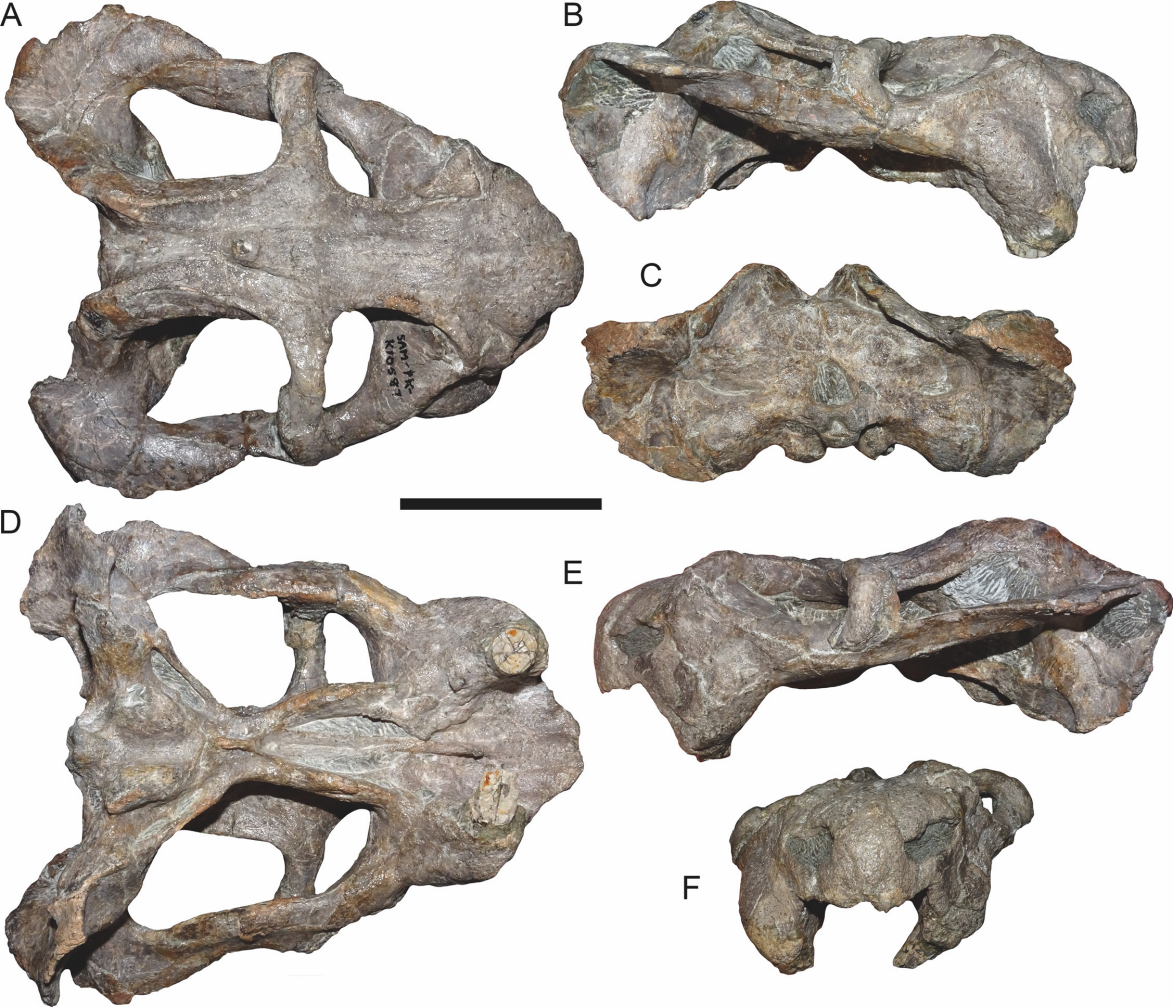
SAM-PK-K10587, referred specimen of *Bulbasaurus phylloxyron* gen. et sp. nov., in (A) dorsal, (B) right lateral, (C) occipital, (D) ventral, (E) left lateral, and (F) anterior views. Scale bar equals 5 cm.

*Referred specimens*: CGP/1/938, a nearly-complete skull from Wilgerbosch Kloof, Fraserburg, Northern Cape Province, South Africa (Figs. 7B, 7E, 7H and 7K); CGP/1/949, a complete skull from Wilgerbosch Kloof, Fraserburg, Northern Cape Province, South Africa (Figs. 8–11); CGP/1/970, a complete skull, lower jaws, and partial postcranium from Blaauwkrans, Beaufort West, Western Cape Province, South Africa (Figs. 12–14); CGP/1/2263, a nearly-complete, anteroposteriorly crushed skull from an uncertain locality (Fig. 15); SAM-PK-K10106, a complete but only partially prepared, dorsoventrally crushed skull and lower jaws from Paalhuisberg, Beaufort West, Western Cape Province, South Africa; SAM-PK-K10587, a small, dorsoventrally crushed skull from Doornhoek, Beaufort West, Western Cape Province, South Africa (Fig. 16). All referred specimens are also from the *Tropidostoma* Assemblage Zone.

*Etymology*: “Leaf razor” (from the latinized Greek *phyllos* and *xyron*), referring to the slicing, inferred keratinous edge of the jaw, used in cutting plant material during feeding. A noun in apposition.

*Diagnosis*: Dicynodont distinguished from all taxa other than geikiids by the combination of a transverse nasofrontal ridge, extremely broad interorbital region, and twisted squamosal contribution to subtemporal bar. Distinguished from the geikiids *Aulacephalodon*, *Pelanomodon*, and *Geikia* by the near-confluence of the nasal bosses (covering the majority of the nasal surface), absence of prefrontal bosses, sharply curved, ‘hook’-like beak tip, tall, highly discrete premaxillary ridge, presence of massive tusks at relatively small skull size, absence of bosses or swellings on the zygomatic arch, relatively weakly twisted subtemporal bar, relatively narrow intertemporal region, and a postparietal out-of-plane with both the occipital plate and skull roof. Further distinguished from *Pelanomodon* and *Geikia* by the presence of tusks and absence of a postcaniniform crest.

## Description

The following description is based primarily on the specimens SAM-PK-K11235 (the holotype), CGP/1/938, and CGP/1/949, all of which are isolated crania of similar size (Table 1). SAM-PK-K11235 is the least complete of the three and has large cracks across the intertemporal bar and snout, but was chosen as the holotype because it is the specimen that best exhibits cranial sutures. Description of the mandible and postcranium is based primarily on CGP/1/970 (one of only two *Bulbasaurus* specimens preserving the mandible and the only specimen preserving any postcrania). All of these specimens were previously identified as the oudenodontid *Tropidostoma* in collections; for this reason contrasts with *Tropidostoma* especially will be emphasized throughout the description, among broader comparisons with known dicynodont taxa. Comparisons with *Tropidostoma dubium* are based primarily on the specimens NHMUK R1662 (a topotype specimen making up part of Seeley’s (1889) original hypodigm for *Tropidostoma*), CGP/1/939, CGP/1/968, SAM-PK-K8603, and SAM-PK-K11238.

The premaxillae of *Bulbasaurus phylloxyron* are fused to form a single median element, as in most dicynodonts (King, 1988; Kammerer & Angielczyk, 2009). On the dorsal skull surface, the premaxilla forms the anterior tip of the ‘beak’; palatally it forms a broad plate making up most of the secondary palate (Figs. 2–5). The tip of the ‘beak’ is sharply deflected in *Bulbasaurus* (Figs. 3, 7H); although not to the extreme degree of the dicynodontoid *Dinanomodon* (Kammerer, Angielczyk & Fröbisch, 2011), it is substantially more ‘hook’-like than in *Tropidostoma* and other cryptodonts (Figs. 7G and 7I). The anterior face of the premaxilla is flattened and bears a highly discrete median ridge with weak depressions on either side (Figs. 2A and 7K). In *Tropidostoma*, although there is usually a median ridge, it is relatively weak and diffuse (Fig. 7J). A well-developed premaxillary ridge is also present in large specimens of *Aulacephalodon*, although it is generally broader and more diffuse at edge than the tall, sharp ridge of *Bulbasaurus*. Posterodorsal to the median ridge, the premaxilla becomes a sharply tapering structure (the ascending process) narrowly extending between the nasal bosses. The posterior extent of the ascending process varies between specimens: it is remarkably long in the holotype, nearly reaching the level of the nasofrontal suture (Fig. 4B), but is relatively short in CGP/1/2263 (Fig. 15A) (although it also nearly reaches the nasofrontal suture in that specimen because of an elongate anterior process of the frontal, which is absent in the holotype). Laterally, the premaxilla contacts the maxilla below the septomaxillary footplate of the external naris (Figs. 2A and 3B). The premaxilla is roughly pentagonal in outline in palatal view (Fig. 5B). Well-developed, paired anterior palatal ridges and a single posterior median ridge are present. The paired anterior palatal ridges are relatively close together, as in *Aulacephalodon* and *Pelanomodon* (Kammerer, Angielczyk & Fröbisch, 2015a). Elongate depressions are present lateral to these ridges, and an additional, median depression is present between them. In *Bulbasaurus*, these three depressions are all of equal depth, as in other geikiids (Fig. 7F) but unlike *Tropidostoma* in which the median depression is substantially deeper than the lateral ones (Fig. 7D). The median ridge is both taller and transversely broader than the anterior ridges. It originates as a low, narrow structure at the posterior edge of the paired anterior ridges, but is not confluent with them (Figs. 5 and 9). It increases in height and breadth posteriorly, with maximal size achieved immediately anterior to the palatine pads. This ridge continues into the interpterygoid region, where it becomes formed by the vomer. Immediately lateral to the median palatal ridge the premaxillary surface is only weakly sloping; there is not a distinct, elongate depression extending along the side of the ridge. However, there are paired premaxillary depressions further laterally, in the region around the anterior terminus of the median ridge (Fig. 5B). Lateral to these depressions are low, rugose, somewhat arcuate ridges near the bases of the caniniform processes. Posteriorly, each of these ridges terminates at a confluence with the anteromedial margin of the palatine pad. Immediately lateral to this confluence is a small but discrete circular fossa at the anterior midpoint of the palatine pad, representing the lateral palatal foramen.

The septomaxilla is a small element largely confined within the external naris (Fig. 2A), but also making up the ventral rim of the naris in the form of a footplate. It is similar in morphology to that of other cryptodonts.

The maxilla is a large bone making up the ventrolateral surface of the snout (Fig. 3B). It bears a well-developed caniniform process housing a massive tusk. The tusks of *Bulbasaurus* are enormous proportional to skull size; in SAM-PK-K11235 the tusk diameter is 1.9 cm on a skull of 14.0 cm dorsal length (see also Table 2). Tusks in *Tropidostoma* are consistently proportionally smaller than those of *Bulbasaurus*, and absolutely smaller in all but the largest known *Tropidostoma* specimens (Table 3). The mean relative tusk diameter (RTD; measured as the ratio of tusk diameter to dorsal skull length) of *Bulbasaurus* is 0.13 (Table 2); by comparison, the mean RTD of *Tropidostoma* is only 0.06 (Table 3). Tusk proportions similar to those of *Bulbasaurus* are otherwise known only in *Aulacephalodon* among cryptodonts. However, *Bulbasaurus* is remarkable in having such large tusks at relatively small skull size. *Aulacephalodon* skulls of comparable size to specimens of *Bulbasaurus* are usually considered juveniles (Tollman, Grine & Hahn, 1980), and have the small, often still-erupting tusks typical of young dicynodonts. *Aulacephalodon* specimens only exhibit adult tusk proportions (i.e., those with RTD >0.10) in skulls greater than 20 cm in length (Table 4), and even these adults tend to have proportionally smaller (mean RTD 0.11) tusks than *Bulbasaurus* (although some individuals attain equivalent proportions). Because of the large size of the tusk root in *Bulbasaurus*, the caniniform process prominently bulges outwards along its lateral surface. The lateral surface of the maxilla is covered in tiny foramina and scattered larger pits. The rugose surface of the entire snout is suggestive of a keratinous covering, as has been argued to be broadly present in dicynodonts (King, 1988; Kammerer, Angielczyk & Fröbisch, 2015b). Dorsally, the maxilla extends between the lacrimal and external naris, before contacting the nasal and anteroventral tip of the prefrontal at its dorsal terminus (Fig. 3B). Posteriorly, it forms an elongate process contributing to the zygomatic arch, underlying first the jugal and then the squamosal before terminating beneath the postorbital bar. The posteroventral face of the caniniform process has a noticeably smoother bone texture than the lateral surface of the maxilla, broken only by two large foramina. No postcaniniform crest is present, unlike all cryptodonts other than *Aulacephalodon* (although in juvenile *Aulacephalodon* of comparable size to *Bulbasaurus*, e.g., BSPG 1934-VIII-516, a postcaniniform crest is present). Posteriorly, there is a straight suture with the jugal and, more medially, an interdigitated suture with the ectopterygoid and palatine (Fig. 5B). A labial fossa is present in this region (Fig. 15D), but the maxilla does not contribute to its border, which is formed by the jugal, ectopterygoid, and palatine.

The nasal forms the roof of the snout and bears a large boss. Paired nasal bosses are present in all known cryptodonts (Kammerer & Angielczyk, 2009) and come in various shapes and sizes within the clade. The nasal bosses of *Tropidostoma* are ovoid and proportionally transversely narrow (Fig. 7A), similar to those of *Oudenodon* and to a lesser extent *Rhachiocephalus*. Additionally, they are clearly separate structures, with unornamented nasal surfaces and a broad, triangular dorsal process of the premaxilla intervening between them. In *Bulbasaurus*, by contrast, the nasal bosses are massive and nearly confluent, with only a narrow strip of premaxilla separating them for most of their length (Figs. 2, 4, 7B and 8). Furthermore the nasal bosses of *Bulbasaurus* are notably rugose, whereas those of *Tropidostoma*, although bearing some pits, have a smoother bone surface. The nasal bosses of *Aulacephalodon* and *Pelanomodon* are also massive and frequently rugose, but unlike *Bulbasaurus* are also separated by broad spans of unornamented nasal (Fig. 7C). A distinct dorsoventral depression extending across the nasal, prefrontal, and maxilla separates the nasal boss from the orbital rim (Fig. 10), as in almost all other cryptodonts. Only in *Odontocyclops* is the nasal boss completely confluent with a prefrontal boss (Angielczyk, 2002). Posterior to the terminus of the ascending process of the premaxilla, the nasals have a short midline suture (Figs. 4B and 15A). This suture is situated in a narrow depression, in the same position where some other dicynodonts have fontanelles during development or neomorphic snout bones (Jasinoski et al., 2014; Kammerer, Angielczyk & Fröbisch, 2015a). Posteriorly, the nasal has a nearly straight but highly interdigitated suture with the frontal. In the holotype this interdigitation is dense but the individual processes are relatively short (Fig. 4), whereas in CGP/1/2263 there is a combination of dense interdigitation and a few large processes (notably an anterior process of the frontals that nearly extends between the nasal bosses; Fig. 15A). This suture is weakly but distinctly raised relative to the surrounding bone. Although not as well-developed as the tall nasofrontal ridge of *Aulacephalodon* and *Pelanomodon*, this raised suture is quite unlike the condition in *Tropidostoma*, in which there is no difference in skull height across the nasofrontal border.

The lacrimal is a narrow, curved bone that on the snout surface only forms the middle part of the anterior orbital margin (Fig. 3B). The lacrimal contribution to the orbital margin bears the best-developed portion of the orbital ridge, which continues onto the anterior portions of the jugal and prefrontal, but is absent around the rest of the orbital rim. Along its anteroventral margin, the lacrimal is excluded from contacting the naris by the dorsal process of the maxilla. Along its anterodorsal margin it has a variable extent between individuals, sometimes contacting the nasal (Fig. 15B) and sometimes being excluded from the nasal by a thin strip of prefrontal (Fig. 3B). Within the orbit, the lacrimal is perforated by a single, large lacrimal foramen.

The prefrontal is also a small bone that is largely limited to the anterior orbital margin (Figs. 3B–4B). Although its anterodorsal surface is somewhat thickened and convex, no distinct prefrontal boss is present (Figs. 4 and 8), unlike the majority of cryptodonts. Indeed, the lateral surface of the prefrontal in front of the orbit is less protruding than that of the lacrimal, as mentioned above.

**Table 1:** Cranial measurements for *Bulbasaurus phylloxyron*.

Specimens	Basal skull length (cm)	Dorsal skull length (cm)	Snout length (cm)	Interorbital least width (cm)	Anterior intertemporal width (cm)	Posterior intertemporal width (cm)	Temporal fenestra length (cm)
CGP/1/938	15.1	13.4	4.2	4.8	3.9	1.6	7.9
CGP/1/949	16.4	13.1	4.5	4.2	4.4	3.9	7.0
CGP/1/970	N/A	16.0	6.6	5.2	5.3	0.6	9.0
CGP/1/2263	11.1	10.9	3.5	2.4	3.5	4.0	6.7
SAM-PK-K10106	N/A	13.4	5.0	4.8	4.2	1.1	8.7
SAM-PK-K10587	11.1	10.4	2.7	2.2	3.6	3.5	6.1
SAM-PK-K11235	16.9	14.0	5.2	4.4	4.0	0.6	9.6
**Mean (all specimens)**	14.1	13.0	4.5	4.0	4.1	2.2	7.9
**Mean (excluding CGP/1/2263 and SAM-PK-K10587)**	16.1	14.0	5.1	4.7	4.4	1.6	8.4
**Standard deviation (all specimens)**	2.8	1.9	1.3	1.2	0.6	1.6	1.3

Specimens	Interpterygoid vacuity length (cm)	Median pterygoid plate width (cm)	Angle of quadrate ramus (left)	Angle of quadrate ramus (right)	Angle between squamosal rami	Dentary symphysis height (cm)	Dentary ramus height (cm)
CGP/1/938	0.8	1.7	53.0	63.0	150.0	N/A	N/A
CGP/1/949	0.9	1.7	N/A	40.0	149.0	N/A	N/A
CGP/1/970	N/A	N/A	N/A	N/A	N/A	9.8	6.3
CGP/1/2263	N/A	N/A	69.0	66.0	144.0	N/A	N/A
SAM-PK-K10106	N/A	N/A	N/A	N/A	N/A	N/A	N/A
SAM-PK-K10587	0.6	1.4	44.0	52.0	150.0	N/A	N/A
SAM-PK-K11235	0.8	1.8	58.0	56.0	146.0	N/A	N/A
**Mean (all specimens)**	0.8	1.7	56.0	55.4	147.8	9.8	6.3
**Mean (excluding CGP/1/2263 and SAM-PK-K10587)**	0.8	1.7	55.5	53.0	148.3	9.8	6.3
**Standard deviation (all specimens)**	0.1	0.2	10.4	10.2	2.7	N/A	N/A

**Table 2:** Variation in tusk size in *Bulbasaurus phylloxyron*.

Specimens	Dorsal skull length (DSL) (cm)	Left tusk diameter (cm)	Right tusk diameter (cm)	Tusk diameter mean (TDM) (cm)	Relative tusk diameter (TDM/DSL)
CGP/1/938	13.4	1.9	2.2	2.1	0.15
CGP/1/949	13.1	1.4	1.7	1.6	0.12
CGP/1/970	16.0	2.2	N/A	2.2	0.14
CGP/1/2263	10.9	1.2	1.3	1.3	0.12
SAM-PK-K10106	13.4	1.8	1.7	1.8	0.13
SAM-PK-K10587	10.4	1.2	1.3	1.3	0.12
SAM-PK-K11235	14.0	2.0	1.8	1.9	0.13
**Mean**	13.0	1.7	1.7	1.7	0.13
**Standard Deviation**	1.9	0.4	0.3	0.4	0.01

**Table 3:** Variation in tusk size in *Tropidostoma dubium*.

Specimens	Dorsal skull length (DSL) (cm)	Left tusk diameter (cm)	Right tusk diameter (cm)	Tusk diameter mean (TDM) (cm)	Relative tusk diameter (TDM/DSL)
CGP/1/930	13.4	1.1	1.1	1.1	0.08
CGP/1/2173	12.1	0.5	0.6	0.5	0.04
CGS CM86-573	17.0	0.9	0.9	0.9	0.05
CGS F7	19.1	1.1	0.7	0.9	0.05
CGS F11	19.9	0.5	1.4	1.0	0.05
CGS R98	13.1	0.9	0.9	0.9	0.07
CGS RMS155	14.9	0.9	1.3	1.1	0.07
CGS RMS213	20.9	1.6	1.8	1.7	0.08
CGS RMS244	14.0	0.5	N/A	0.5	0.03
CGS RMS631	18.8	0.9	0.8	0.8	0.04
CGS RS327	22.4	1.9	1.5	1.7	0.08
CGS RS538	13.9	0.6	0.8	0.7	0.05
NHMUK R1662	25.3	2.1	1.5	1.8	0.07
NHMUK R4048	19.1	1.7	1.7	1.7	0.09
SAM-PK-K6742	23.4	N/A	1.4	1.4	0.06
SAM-PK-K6808	18.0	0.5	N/A	0.5	0.03
SAM-PK-K6940	14.1	0.5	0.3	0.4	0.03
SAM-PK-K8603	17.7	1.1	1.0	1.1	0.06
SAM-PK-K8639	16.2	0.9	0.4	0.7	0.04
SAM-PK-K10681	16.8	1.7	1.5	1.6	0.09
SAM-PK-K11015	13.0	1.1	1.2	1.1	0.09
SAM-PK-K11183	22.3	1.5	1.3	1.4	0.06
SAM-PK-K11238	13.6	1.0	1.0	1.0	0.07
SAM-PK-K11255	21.8	2.3	1.0	1.6	0.08
**Mean**	17.5	1.1	1.1	1.1	0.06
**Standard deviation**	3.8	0.5	0.4	0.4	0.02

**Table 4:** Variation in tusk size in *Aulacephalodon bainii*.

Specimens	Dorsal skull length (DSL) (cm)	Left tusk diameter (cm)	Right tusk diameter (cm)	Left/right tusk diameter mean (TDM) (cm)	Relative tusk diameter (TDM/DSL)
BP/1/300	18.2	0.6	0.8	0.7	0.04
BP/1/304	40.0	4.4	3.9	4.2	0.10
BP/1/2460	34.2	3.8	3.0	3.4	0.10
BP/1/2983	36.9	4.8	3.9	4.3	0.12
BP/1/4087	25.3	3.5	3.1	3.3	0.13
BSPG 1934-VIII-516	14.5	0.9	0.9	0.9	0.06
CGP/1/268	22.6	2.5	2.4	2.5	0.11
CGP/1/657	26.4	3.0	3.0	3.0	0.11
CGP/1/740	28.7	3.4	2.8	3.1	0.11
CGS S6	24.5	2.8	3.3	3.1	0.12
SAM-PK-3423	22.7	2.7	2.8	2.7	0.12
SAM-PK-5862	15.2	0.9	0.8	0.9	0.06
SAM-PK-10021	33.9	3.9	3.8	3.8	0.11
SAM-PK-10048	18.6	1.3	1.3	1.3	0.07
SAM-PK-10673	25.1	2.7	2.4	2.6	0.10
USNM 24621	37.0	3.2	4.3	3.7	0.10
**Mean (all specimens)**	26.5	2.8	2.7	2.7	0.10
**Mean (specimens with >20 cm skull length)**	29.8	3.4	3.2	3.3	0.11
**Standard deviation**	8.0	1.3	1.1	1.2	0.03

The jugal is an elongate bone that is primarily part of the zygoma (Fig. 4B). It has a short facial exposure above the posterior portion of the caniniform process and makes up the ventral wall of the orbit (Fig. 3B). As previously described, it also borders the ventral surface of the maxilla, behind the caniniform process (Fig. 5B), where it partially surrounds the labial fossa. From here, the jugal curves posterolaterally, forming the ventromedial edge of the zygoma until reaching the end of the temporal fenestra. It also has a short contribution to the dorsal surface of the subtemporal bar, immediately behind the postorbital bar, and also forms part of the posteromedial face of the postorbital bar. It is generally similar in morphology to that of other cryptodonts (although *Pelanomodon* is aberrant in having a jugal boss as part of its contribution to the postorbital bar; Kammerer, Angielczyk & Fröbisch, 2015a).

The zygomatic ramus of the squamosal tapers anteriorly, separating the maxilla and jugal below the postorbital bar before terminating below the orbital midlength (Fig. 3B). The squamosal is prominently twisted in the subtemporal bar, such that what was its lateral surface anteriorly becomes ventral surface posteriorly. Some deflection of the edge of the squamosal in the posterior part of the subtemporal bar is typical in cryptodonts (including *Tropidostoma*; Fig. 7G), but in the large geikiids this is taken to an extreme, with complete reversal of the lateral and medial faces of the zygoma along its length (Fig. 7I). *Bulbasaurus* exhibits an intermediate condition, with some twisting of the zygoma (Fig. 7H) but not to the degree seen in *Aulacephalodon* and *Pelanomodon*. The squamosal flares broadly posterolaterally around the temporal fenestra before curving inwards to form a short intertemporal ramus immediately behind the postorbital (Fig. 4B). Posteriorly, the squamosal is a major contributor to the occipital plate, bordering the tabular and fused supraoccipital-periotic element laterally (Fig. 6B). Medial extensions of the squamosal surround the lateral half of the post-temporal fenestra, as in several other cryptodont taxa (*Aulacephalodon*, *Pelanomodon*, *Oudenodon* (Kammerer, Angielczyk & Fröbisch, 2015a), and *Tropidostoma* (C Kammerer, pers. obs., 2016)) A mediolaterally deep, dorsoventrally narrow notch is present between the zygomatic and quadrate rami of the squamosal (Fig. 11).

The frontal is largely restricted to the interorbital region in *Bulbasaurus* (Fig. 4B). It has a rugose edge where it forms part of the dorsal margin of the orbit, and several deep pits on its dorsal surface. The frontal curves downwards posteromedially, forming a deep median depression with the preparietal between the postorbital bars, anterior to the pineal foramen (Figs. 4 and 8). A depression in this part of the skull is sometimes weakly developed in *Tropidostoma* and *Oudenodon*, but is absent in other geikiids. This is probably related to the general broadening and flattening of the intertemporal region in these taxa relative to most other bidentalians. Posterolaterally, the border between the frontal and postorbital forms a sharp ridge. A distinct postfrontal appears to be absent in *Bulbasaurus*(Fig. 4B). The postfrontal is a large, triangular element at the posterodorsal margin of the orbit in *Tropidostoma* (Fig. 7A) and is usually narrower but still distinct in *Oudenodon*. The postfrontal is not discernible in adult geikiids such as *Aulacephalodon*, although it appears to be present in some juvenile specimens (e.g., BSPG 1934-VIII-516), suggesting that it fuses with the postorbital during growth. The absence of a distinct postfrontal in *Bulbasaurus* is further evidence of ‘adult’ geikiid characters being present at small skull size in this taxon.

The postorbital makes up the majority of the postorbital bar and the medial edge of the temporal fenestra (Figs. 3B, 4B and 8). The postorbital bar is typically thick in cryptodonts, but is particularly robust in geikiids, *Bulbasaurus* included. Unlike other geikiids, however, the postorbital bar of *Bulbasaurus* does not bear any bosses or dorsoventral ridges. The posterior ramus of the postorbital has a very steep lateral face, with a sharp break in slope between the postorbital laterally and the skull roof (frontal and parietal) medially. The lateral face of the postorbital is distinctly concave and would have served as the attachment site for jaw musculature. The posterior rami of the postorbitals converge posteriorly, creating a ‘pinched’ intertemporal bar. The degree of this convergence varies between specimens; in SAM-PK-K11235, the postorbitals almost completely overlap the parietals posteriorly, with only a narrow strip of parietal exposed between them (Fig. 4), whereas in CGP/1/938 and CGP/1/949, a trough-like median exposure of the skull roof is retained in the posterior intertemporal bar (Figs. 7B and 8). This style of variability is common in *Tropidostoma* and *Oudenodon*; in adult specimens of *Aulacephalodon* and *Pelanomodon* the parietal always remains broadly exposed in the intertemporal bar. Generally, however, postorbital-parietal overlap in *Tropidostoma*, when present, occurs throughout the length of the intertemporal bar (Fig. 7A) instead of in a ‘pinched’ posterior span. The posterior ramus of the postorbital terminates just beyond the point of the occipital plate (Fig. 4B).

The preparietal is a small, median element anterior to the pineal foramen (Fig. 4B). It is weakly depressed relative to the surrounding bone, taking into account that the entire surrounding region is strongly depressed (as discussed in the description of the frontal above). Preparietal depression is common in cryptodonts, and is also present in *Tropidostoma*. However, in *Tropidostoma* this bone is typically a narrow, anteroposteriorly elongate element with a pointed anterior tip (Fig. 7A). In *Bulbasaurus*, the preparietal is relatively wide, with a broadly rounded anterior tip (Fig. 4B), as in *Aulacephalodon* and *Pelanomodon*. The pineal foramen is a small, subcircular opening situated between the parietals (posteriorly and laterally) and preparietal (anteriorly). It is not elevated on a mound-like boss (as in rhachiocephalids, *Endothiodon*, and some large specimens of *Aulacephalodon*), but does have a raised, collar-like rim (Fig. 8). As noted above, the parietals are largely obscured by the postorbitals within the intertemporal bar in several specimens of *Bulbasaurus*. When exposed, their surface is weakly concave and otherwise unornamented.

As is usual in dicynodonts, the vomer is exposed only within the interpterygoid vacuity in ventral view (Fig. 5B). Anteriorly it is a narrow, rod-like element confluent with the median palatal ridge of the premaxilla. Posteriorly is slopes dorsally and bifurcates, developing a median trough before terminating in paired, flattened rami pressed against the medial walls of the anterior pterygoid rami.

The palatine is exposed ventrally in the form of a palatine pad, a roughly teardrop-shaped structure that would have formed part of the masticatory surface of the palate, and a shelf-like portion posterolateral to the pad that braces the medial edge of the anterior pterygoid ramus (Fig. 5B). The transversely expanded portion of the palatine pad is highly rugose, indicative of keratinous covering. Anterior to this is a short, sloping stretch of palatine contacting the premaxilla, with smooth bone texture. The palatine shelf attenuates posteriorly, extending along the medial wall of the pterygoid; this portion of the bone also has smooth bone texture. Although attenuate, this posterior portion is relatively thick in *Bulbasaurus* (Figs. 5B, 7E) compared to that of *Tropidostoma* (Fig. 7D) or *Oudenodon*, but is comparable to that of *Aulacephalodon* (Fig. 7F). In addition to its palatal contribution, the palatine is also exposed posterolaterally, behind the jugal and above the anterior pterygoid ramus along the rim of the subtemporal fenestra. Here, it contributes to the posterior rim of the labial fossa.

The ectopterygoid is a small, laminar element bordering the lateral wall of the anterior pterygoid ramus (Figs. 3B and 5B). It curves slightly laterally anteriorly, where it forms part of the border of the labial fossa. The anterior ramus of the pterygoid is a robust structure in *Bulbasaurus*, as in other geikiids. In *Tropidostoma*, the anterior pterygoid ramus is relatively narrower, with weaker lateral splay, and is generally more elongate (Fig. 7D). Thin crests on the ventral edges of the anterior pterygoid rami converge into the crista oesophagea posteriorly, on the median pterygoid plate (Fig. 9). The crista oesophagea is very well developed in *Bulbasaurus*, forming a tall, blade-like structure. Finally, the quadrate ramus of the pterygoid is a rod-like process, highly splayed outwards (57° relative to the long axis of the skull in the holotype) to contact the quadrate posterolaterally.

Sutures in the braincase of *Bulbasaurus* are not evident in any of the known specimens; it is likely that the braincase elements are extensively fused. Posterior to the median pterygoid plate, diverging ridges on what should be the parabasisphenoid (by comparison with other dicynodonts; King, 1988) extend towards the basal tubera (Fig. 5B). Each tuber is a thick, crescentic structure surrounding a ventrolateral exposure of the fenestra ovalis. The stapes is preserved in the specimen CGP/1/2263 (Fig. 15D). It is a stout, dumbbell-shaped element similar to those of other cryptodonts. A deep depression separates the basal tubera, and no intertuberal ridge is present. Posterior to this depression is the occipital condyle, which has the typical tripartite morphology of dicynodonts (presumably composed of a ventral basioccipital portion and paired dorsal exoccipital portions, although these elements are completely fused in *Bulbasaurus*). Circular jugular foramina are present ventrolateral to the occipital condyle, as is also typical of dicynodonts (Fig. 5B). Dorsal to the occipital condyle, the foramen magnum is ovoid, taller than wide (Figs. 6B and 11).

The occiput of SAM-PK-K11235 is well-prepared and fairly well-preserved (other than the broken edges of the squamosals), and clearly shows that most of the occipital elements are fused (Fig. 6B). Fusion between the opisthotic and prootic to form a periotic bone is common in dicynodonts, and incorporation of additional braincase bones is also observed in many taxa (Kammerer, Angielczyk & Fröbisch, 2015a). In *Bulbasaurus*, the occipital/braincase element is formed from fusion of the supraoccipital, exoccipitals, basioccipital, opisthotic, and prootic. The supraoccipital portion of this fused element is unusually expansive, making up the majority of the non-squamosal area of the occiput above the foramen magnum. The supraoccipital is typically a large element in dicynodonts, but usually the postparietal is nearly equal in size and the occiput has substantial contributions by the tabulars (this is the condition in *Tropidostoma*, for instance). In *Bulbasaurus*, the postparietal and tabulars are substantially smaller than the supraoccipital. The tabular is an arcuate, paired element between the squamosal, supraoccipital, and postparietal. Unusually for a therapsid, its long axis is horizontal rather than vertical. The most unusual occipital element is the postparietal, a median element at the top of the occipital plate. In most dicynodonts the postparietal is a flat, laminar element in-plane with the rest of the occiput, although it may have a dorsal process extending onto the skull roof (prominently in oudenodontids and especially so in cistecephalids). In *Bulbasaurus*, however, the entire postparietal is out-of-plane with both the skull roof and occipital plate, instead forming a sharply-angled ‘divot’ in the back of the skull. A very strongly developed nuchal crest is present along the postparietal midline and restricted entirely to that element.

The mandible is only preserved in two specimens of *B*. *phylloxyron*: CGP/1/970 (Figs. 12–14) and SAM-PK-K10106, and is largely unprepared in the latter. In CGP/1/970, the mandible is partially occluded to the cranium, but is somewhat dislocated (Fig. 14), revealing details of its dorsal surface (Fig. 13). In general, the mandible is similar to that of *Aulacephalodon*. The dentaries are fused to form an edentulous beak. The anterior face of the jaw symphysis is smoothly convex, without an anterior median ridge (Fig. 14). A sharp ridge delimits the edge between the anterior face of the symphysis and the lateral face of the jaw ramus (Fig. 12B), which is also present in *Pelanomodon* and *Geikia* but not *Aulacephalodon*. The anterodorsal terminus of the jaw comes to a sharp, pointed tip with a concave posterior surface. Although lower in dorsoventral height than the symphysis, the lateral portion of the jaw ramus is still tall and robust. The mandibular fenestra is a narrow, oval opening at mid-height on the dentary ramus; it is directly overhung by a small lateral dentary shelf that expands anterodorsally to form a broadened, diffuse boss. Unfortunately, the postdentary bones are not well-preserved in CGP/1/970; what is present is indistinguishable from that of other geikiids. Ventrally, the right articular is partially exposed, and has a well-developed, bulbous retroarticular process (Fig. 14).

The postcranial elements preserved in CGP/1/970 consist of a partially disarticulated set of cervical vertebrae, ribs, pectoral girdle, and forelimb elements (Figs. 13 and 14). Of these elements only the ribs (Fig. 13) and a humerus (Fig. 14) are reasonably exposed. The proximal ends of two left and four right ribs are preserved. They are bicipital and gently curved along the shaft, as in other dicynodonts. The humerus appears to represent a right humerus (visible as the large element at the bottom of Fig. 14). Its distal tip is broken off but it is otherwise intact, albeit extensively obscured by matrix and other bones. The deltopectoral crest is robust and strongly separated from the humeral shaft at a perpendicular angle.

## Discussion

### Ontogeny of *Bulbasaurus*

Most specimens of *Bulbasaurus phylloxyron* occupy the size range of 13–16 cm dorsal skull length. Two specimens fall outside of this range: CGP/1/2263 (Fig. 15) at 10.9 cm and SAM-PK-K10587 (Fig. 16) at 10.4 cm dorsal skull length. The short skull of CGP/1/2263 is at least partially artifactual, as this specimen is strongly anteroposteriorly compressed. SAM-PK-K10587, however, represents a genuinely smaller skull (in all dimensions) than other specimens of *Bulbasaurus*. Intriguingly, this specimen also differs from the larger specimens in a suite of cranial features, providing insight into the possible ontogenetic trajectory of *Bulbasaurus*.

Although the snout tip of SAM-PK-K10587 is still noticeably deflected (Figs. 16B and 16E), the snout in general is shorter and less ‘hook’-like than in the larger specimens of *Bulbasaurus*. The tusks of SAM-PK-K10587 are large and already-erupted (Fig. 16D), unlike *Aulacephalodon* or *Tropidostoma* skulls of equivalent size, but are on the low end of tusk proportions for *Bulbasaurus* (Table 2). The nasal bosses are weakly developed (Fig. 16A) and more clearly separated by a wider intervening ascending process of the premaxilla (Fig. 16F) than in the larger specimens. The interorbital region is proportionally narrower than in larger specimens, whereas the intertemporal region is comparatively broader (Fig. 16A). Although the curvature of the intertemporal region is already evocative of having a posterior ‘pinched’ portion in SAM-PK-K10587, no overlap of the postorbital on the parietal is present. There is also relatively little depression of the interorbital and intertemporal region, with the preparietal even being somewhat raised relative to the surrounding flat frontoparietal region (Fig. 16A). Finally, the zygomatic ramus of the squamosal is only slightly deflected at its posterodorsal edge; the subtemporal bar is not twisted 90°(Figs. 16B and 16E).

Most of the differences between SAM-PK-K10587 and the larger *Bulbasaurus* skulls are also observed in the ontogeny of *Aulacephalodon* (Tollman, Grine & Hahn, 1980; Kammerer, Angielczyk & Fröbisch, 2015a). The main exception is in the width of the intertemporal bar, which seems to follow the ancestral cryptodont pattern in *Bulbasaurus* (increasing overlap of the parietals by the postorbitals with growth, as is also observed in oudenodontids and rhachiocephalids) rather than the geikiid pattern (expansion of intertemporal parietal width with growth; Kammerer, Angielczyk & Fröbisch, 2015a). Remarkably, development of the complete suite of ‘adult’ geikiid features in *Bulbasaurus* occurs between skulls differing only in ∼3 cm length, whereas in *Aulacephalodon* this degree of ontogenetic change is observed between skulls differing in 10 cm length or more. Presence of these features in ∼13 cm long cranial specimens of *Bulbasaurus* suggests that most ‘adult’ characteristics of geikiids were evolved at small size and retained by giant descendants, not evolved in concert with the adoption of large body size.

### Comparisons with other nominal cryptodont species

Twentieth century Karoo therapsid workers, especially Robert Broom, established a superfluity of dicynodont taxon names, the majority of which have subsequently been synonymized (King, 1988; Wyllie, 2003; Kammerer, Angielczyk & Fröbisch, 2011). Most of these synonymies have proven uncontroversial, particularly in the cases of supposed sympatric ‘species’ whose holotypes are distinguished solely by degree of taphonomic distortion. However, recent studies of dicynodont anatomy and stratigraphic distribution have revealed that the latter-day spate of taxonomic lumping may have been a bit overzealous, and that some previously-synonymized taxa are better recognized as distinct morphospecies (e.g., Angielczyk, 2002; Kammerer, Angielczyk & Fröbisch, 2015b). Given the vast array of available names for Karoo dicynodonts, it is important to consider whether the novel morphotype recognized herein could belong to a preexisting nominal species. Comparisons with well-known, valid species such as *Tropidostoma dubium* and *Aulacephalodon bainii* have already been presented in the context of the Description, but in this section, additional attention will be given to nominal species currently lurking in synonymy lists and *species inquirenda*.

**Figure 17:**
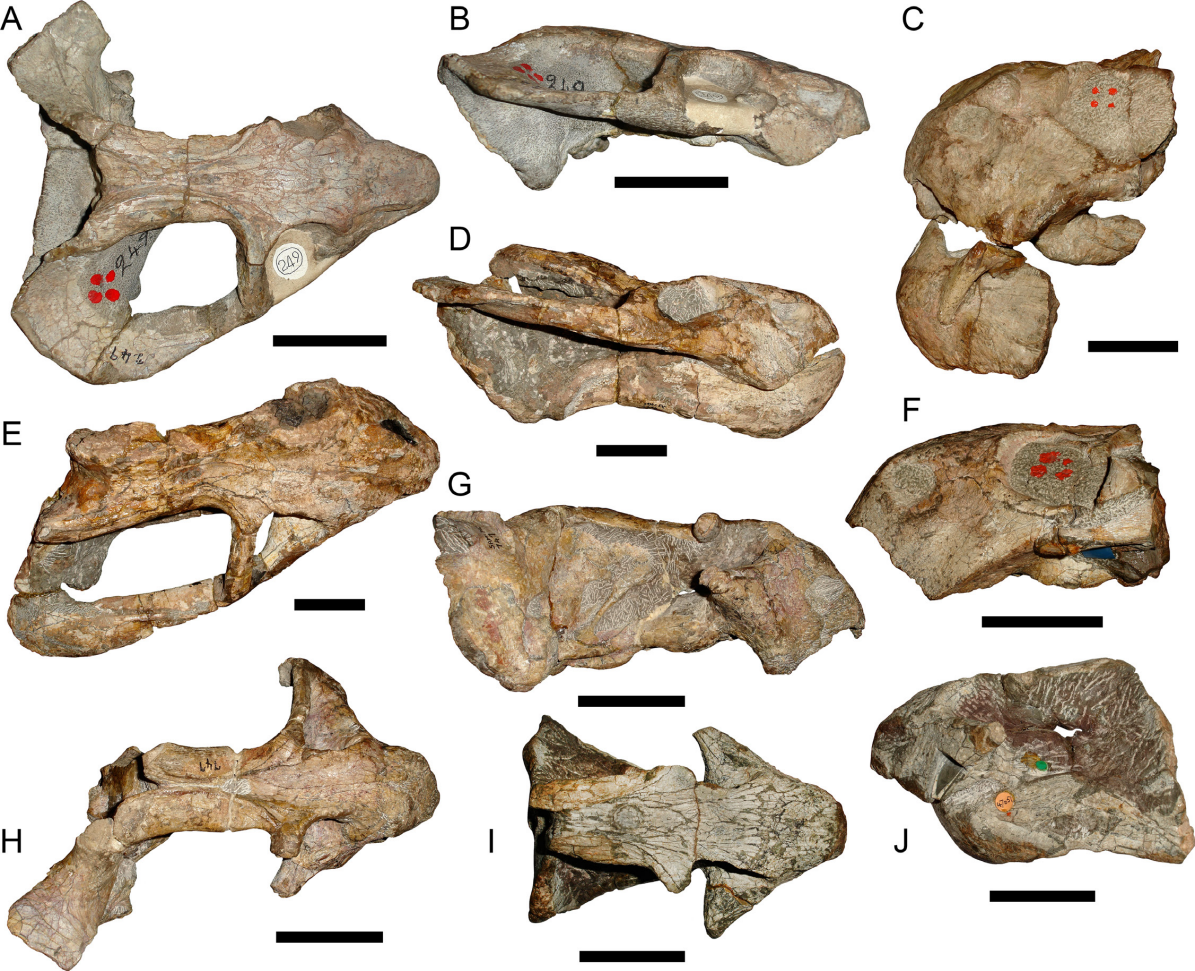
Holotypes of nominal species currently considered synonymous with *Tropidostoma dubium*. TM 249, holotype of *Cteniosaurus platyceps* Broom, 1935, in (A) dorsal and (B) right lateral view. TM 252, holotype of *Dicynodon validus* Broom, 1935, in (C) left lateral view. SAM-PK-2356, holotype of *Dicynodon rogersi* Broom & Haughton, 1917, in (E) dorsal and (D) right lateral view. TM 250, holotype of *Dicynodon acutirostris* Broom, 1935, in (F) left lateral view. SAM-PK-747, holotype of *Dicynodon cavifrons* Broom & Haughton, 1917, in (H) dorsal and (G) right lateral view. NHMUK 47051, holotype of *Dicynodon dubius* Owen, 1876, in (I) dorsal and (J) left lateral view. Scale bars equal 5 cm.

Prior to the recognition of *Bulbasaurus phylloxyron* as a distinct morphotype, all of the specimens of this taxon were identified in collections as *Tropidostoma*. As such, the most likely source for potential preexisting names representing this morphotype is among the nominal species currently considered synonymous with *Tropidostoma dubium* (Keyser, 1973; Botha & Angielczyk, 2007; Kammerer, Angielczyk & Fröbisch, 2011). Seven nominal dicynodont species are currently in synonymy with *T. dubium*: *Tropidostoma dunnii* Seeley, 1889 (the type species of *Tropidostoma*), *Dicynodon microtrema* Seeley, 1889, *Dicynodon cavifrons* Broom & Haughton, 1917, *Dicynodon rogersi* Broom & Haughton, 1917, *Cteniosaurus platyceps* Broom, 1935, *Dicynodon acutirostris* Broom, 1935, and *Dicynodon validus* Broom, 1935. Within the current hypodigm for *Tropidostoma dubium*, two cranial morphs can be recognized: one ‘robust’ with a relatively tall, short snout and large tusks, and one ‘gracile’ with a relatively long, low snout and small tusks. The holotypes of *Dicynodon dubius* (Figs. 17I, 17J), *D. cavifrons* (Figs. 17G, 17H), and *D*. *validus* (Fig. 17C) correspond to the ‘robust’ morph, whereas the holotypes of *C*. *platyceps* (Figs. 17A, 17B), *D*. *rogersi* (Figs. 17D, 17E), and *D*. *acutirostris* (Fig. 17F) correspond to the ‘gracile’ morph. It is possible that this morphological disjunct reflects taxonomic distinction, but given that similar morphs are seen in the confamiliar *Oudenodon bainii* (C Kammerer, pers. obs., 2016) it is more likely that this represents sexual dimorphism, a not-uncommon occurrence in dicynodonts (Tollman, Grine & Hahn, 1980; Sullivan, Reisz & Smith, 2003; Ray, 2005; Kammerer, Angielczyk & Fröbisch, 2015a; Kammerer, Angielczyk & Fröbisch, 2015b). Whether these morphs exhibit the frequency expected for sexual dimorphism and whether they are truly dimorphs or represent a cline of individual variation will be addressed in a future contribution. Importantly for the topic at hand, however, neither of these morphs corresponds to the morphology of *Bulbasaurus*.

The holotypes of *T*. *dunnii* (NHMUK R866) and *D*. *microtrema* (NHMUK R868) consist solely of isolated occipital plates with attached intertemporal bars. Although the incompleteness of these specimens limits comparisons, they can clearly be differentiated from *Bulbasaurus* by their intertemporal morphology: both specimens have elongate intertemporal bars in which the postorbitals almost completely overlap the parietals for the extent of their length (similar to that of Fig. 7A). By contrast, in *Bulbasaurus* postorbital overlap of the parietals is limited to a posterior ‘pinched’ portion of the intertemporal bar (Fig. 4) if it is present at all. The holotype of *D*. *cavifrons* (SAM-PK-747) is a well-preserved partial skull missing all of the the left and most of the right temporal arches. This specimen is roughly similar to *Bulbasaurus* in having relatively large tusks and a deep snout (Fig. 17G), characters shared with other ‘robust’ morph *Tropidostoma* specimens. However, it can be distinguished from *Bulbasaurus* by the elongate intertemporal bar, with almost complete postorbital-parietal overlap along its length, the presence of large, triangular postfrontals, a relatively narrow interorbital region, small, ovoid nasal bosses restricted to overhanging the external nares (Fig. 17H), the presence of postcaniniform crests, and thin quadrate rami of the pterygoids. The same features also distinguish the holotypes of *D*. *rogersi* (SAM-PK-2356, a dorsoventrally compressed partial skull missing the left temporal arch) and *C*. *platyceps* (TM 249, a partial skull missing most of the left temporal arch) from *Bulbasaurus*, with the exception of there being a broad intertemporal exposure of the parietal in these specimens (Figs. 17A and 17E). These specimens can be further differentiated from *Bulbasaurus* by their transversely narrow snouts (Figs. 17A and 17E) and relatively small tusks. The holotype of *D*. *acutirostris* (TM 250, an isolated snout) also has a transversely narrow snout, but further differs from *Bulbasaurus* in the complete absence of tusks (Fig. 17F). The holotype of *D*. *validus* (TM 252, a partial snout with the anterior portion of the lower jaws) is another ‘robust’ morph skull, but can readily be distinguished from *Bulbasaurus* by nasal boss morphology, with a highly discrete ovoid boss restricted to the posterodorsal edge of the external naris (Fig. 17C). Finally, it is worth making comparisons to the holotype of *Tropidostoma dubium* (formerly *Dicynodon dubius*) itself, as the contrasts with *T*. *dubium* in the Description were based on exemplary referred specimens rather than the type. The holotype (NHMUK 47051) is a partial skull and occluded lower jaws, missing the temporal arches. Very large, triangular postfrontals are present in this specimen, and the nasal bosses exhibit the morphology typical for *Tropidostoma* (Figs. 17I and 17J). Furthermore, in this specimen the tusks were just beginning to erupt at the time of death (visible within the ground-off caniniform process; see Fig. 17J), despite being a larger individual (dorsal skull length: 11.1 cm) than SAM-PK-K10587, which although the smallest (dorsal skull length: 10.4 cm) specimen of *Bulbasaurus* already has large, fully-erupted tusks (Fig. 16).

Few specimens comparable to *Bulbasaurus* are present among the type series of other synonym-rich cryptodonts. The many synonyms of *Oudenodon bainii* can be distinguished from *Bulbasaurus* by their consistent lack of tusks, in addition to various shared oudenodontid characters that also distinguish *Tropidostoma* from *Bulbasaurus* (such as general gracility of the pterygoids and intertemporal morphology). Almost all holotypes referable to *Aulacephalodon bainii* are large specimens exhibiting the full suite of features that distinguish adults of that species from *Bulbasaurus* (e.g., extremely broad intertemporal region, massive but discrete nasal bosses protruding over the external nares, bosses or swellings on the zygomatic arch). A notable exception, however, is BP/1/763, a dorsoventrally compressed complete skull that forms the holotype of *Proaulacocephalodon miltoni* Toerien, 1955. Tollman, Grine & Hahn (1980) considered BP/1/763 to represent a juvenile individual of *Aulacephalodon bainii*, and attributed its very small tusks, narrow intertemporal and interorbital regions, and lack of nasal and prefrontal bosses to an early stage of ontogeny. This specimen is indeed relatively small (dorsal skull length: 15.6 cm) compared with most skulls of *A*. *bainii*. However, the recent discovery of even smaller specimens (notably SAM-PK-K11409, dorsal skull length: 10.2 cm; formerly B 168, see Kammerer, Angielczyk & Fröbisch, 2015a) referable to *A*. *bainii* that already show distinct, protruding nasal bosses and intertemporal morphology similar to that of adult *Aulacephalodon* suggests that these features cannot be explained by ontogeny in BP/1/763. The proper attribution of BP/1/763 is uncertain at present, but it is probably not referable to *Aulacephalodon*. It is also not conspecific with *Bulbasaurus phylloxyron*, being distinguished by the proportionally shorter intertemporal region, narrower interorbital region, absence of nasal bosses, extremely small tusks (despite being an absolutely larger skull than nearly all specimens of *Bulbasaurus*), and relatively thin quadrate rami of the pterygoids. A similarly problematic specimen is TM 1480, holotype of *Dicynodon hartzenbergi* Broom, 1940. This specimen consists of a skull and lower jaws, with the anterior tip of the snout worn off. Brink (1986) considered TM 1480 to be a juvenile of *Aulacephalodon*, but the absence of protruding nasal bosses and tusks makes this identification unlikely. Kammerer, Angielczyk & Fröbisch (2011) noted some similarities with geikiids, but left this taxon as a species of uncertain attribution. In overall skull proportions this specimen is similar to that of *Bulbasaurus*, but differs from the latter taxon in the absence of tusks, substantially narrower interorbital region, and apparent restriction of the small nasal bosses to the edges of the external nares. In these characters it is similar to *Oudenodon*, and this specimen may eventually prove to be an anteroposteriorly-deformed specimen of *O*. *bainii*, although further research is needed. In addition to the morphological features listed above, from a stratigraphic standpoint it is unlikely that either *Proaulacocephalodon miltoni* or *Dicynodon hartzenbergi* are conspecific with *Bulbasaurus phylloxyron*, because neither are from the *Tropidostoma* AZ, both hailing from the overlying *Cistecephalus* AZ (with BP/1/763 found in Windpoort, Murraysburg, Western Cape Province, and TM 1480 found in Petersburg, Graaff-Reinet, Eastern Cape Province).

### Relationships of *Bulbasaurus*

*Bulbasaurus phylloxyron* was included in a modified version of the anomodont phylogenetic analysis of Kammerer, Angielczyk & Fröbisch (2011), incorporating data from the revisions of Castanhinha et al. (2013), Kammerer, Angielczyk & Fröbisch (2013), Kammerer, Angielczyk & Fröbisch (2015b), Kammerer, Bandyopadhyay & Ray (2016), Angielczyk et al. (2016), and Boos et al. (2016). This analysis includes 21 continuous characters, 153 discrete state characters, and 103 operational taxonomic units. Codings for *Bulbasaurus* were based on all known specimens, with variation between specimens coded as polymorphism and continuous characters coded as means (see Data S1). Analyses were run in TNT v1.1 (Goloboff, Farris & Nixon, 2008) using New Technology search parameters (sectorial search, parsimony ratchet, drift, and tree fusing) with an initial driven search level of 65 and the requirement of finding the most parsimonious tree 20 times. Bootstrap support was calculated based on 10,000 replicates.

**Figure 18:**
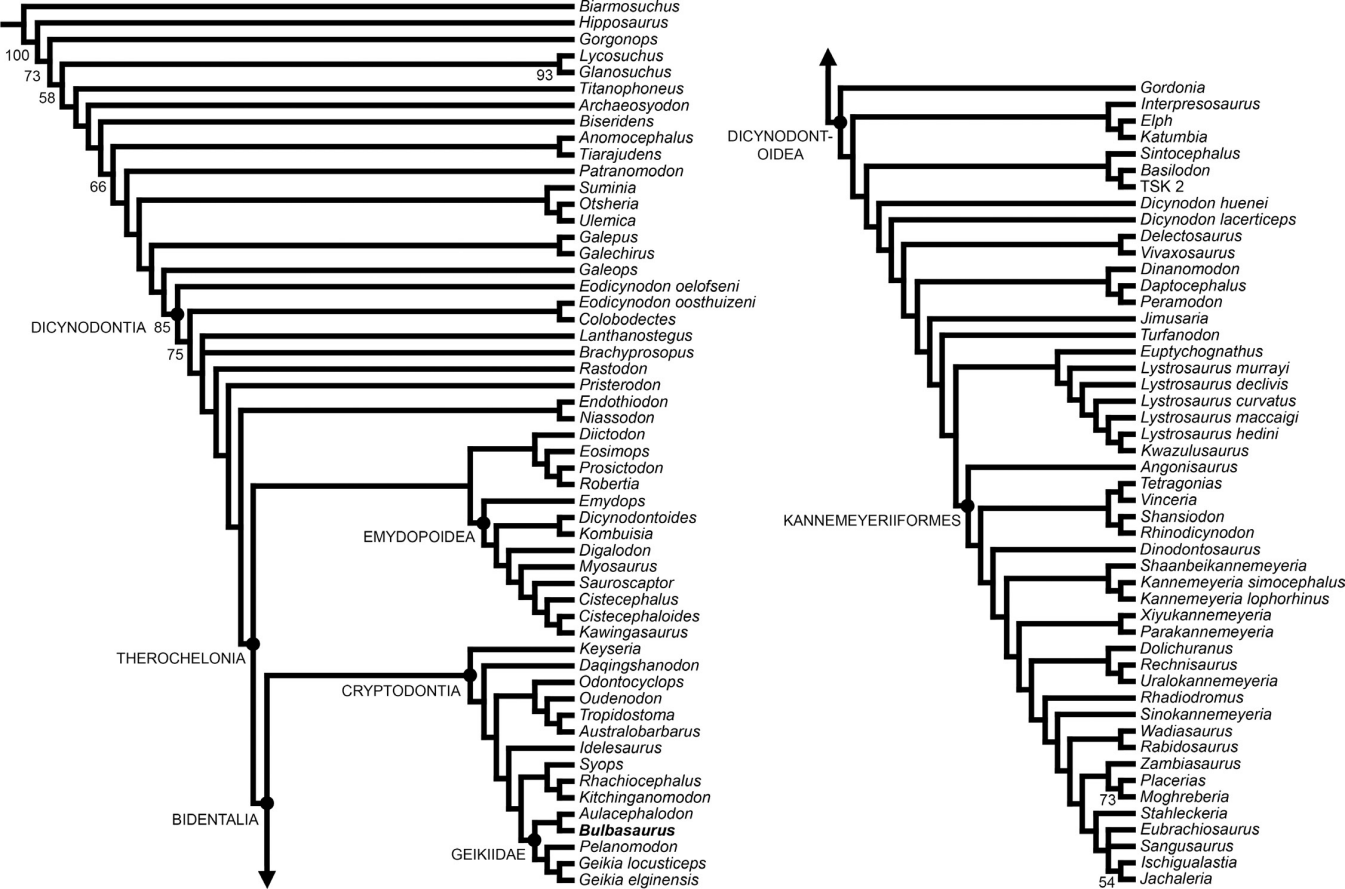
Strict consensus of the two most parsimonious trees resulting from the phylogenetic analysis. Major clades discussed in the text are labeled at their respective nodes; numbers at nodes represent bootstrap values >50.

Two most parsimonious trees of length 1032.706 were recovered, with a consistency index of 0.234 and a retention index of 0.709 (Fig. 18). The only differences between the two trees are in the positions of the middle Permian taxa *Brachyprosopus broomi* and *Lanthanostegus mohoii* (which swap positions as successive outgroups to the clade containing *Rastodon*, *Pristerodon*, Endothiodontia, and Therochelonia) and the Triassic stahleckeriids *Eubrachiosaurus* and *Sangusaurus* (which swap positions as the sister-taxon of *Ischigualastia* + *Jachaleria*). The current tree differs from that of the recent analysis by Boos et al. (2016) in several important regards. Boos et al. (2016) recovered the newly-described middle Permian Brazilian dicynodont *Rastodon* as the earliest-diverging member of Bidentalia. In the current analysis *Rastodon* is recovered in a far more rootward position, as the sister-taxon to all dicynodonts other than *Eodicynodon*, *Colobodectes*, *Brachyprosopus*, and *Lanthanostegus*. Pylaecephalidae (the family containing *Diictodon* and its close relatives) is recovered within Therochelonia, as the sister-taxon to Emydopoidea, as in the majority of recent analyses (Kammerer, Angielczyk & Fröbisch, 2011; Kammerer, Angielczyk & Fröbisch, 2013; Kammerer, Angielczyk & Fröbisch, 2015b; Kammerer, Bandyopadhyay & Ray, 2016; Castanhinha et al., 2013; Cox & Angielczyk, 2014; Angielczyk et al., 2016). By contrast, Boos et al. (2016) recovered Pylaecephalidae outside of Therochelonia, as in a number of earlier analyses (Angielczyk, 2002; Angielczyk, 2007; Angielczyk & Kurkin, 2003; Fröbisch, 2007; Fröbisch & Reisz, 2008; Angielczyk & Rubidge, 2013).

Cryptodontia in its traditional sense (Kammerer & Angielczyk, 2009) was recovered as monophyletic (contra other recent iterations of this analysis; e.g., Boos et al., 2016; Kammerer, Bandyopadhyay & Ray, 2016). *Keyseria* and *Daqingshanodon* were recovered as the earliest-diverging cryptodonts, as in all other analyses in which Cryptodontia is monophyletic (e.g., Kammerer, Angielczyk & Fröbisch, 2011; Kammerer, Angielczyk & Fröbisch, 2013; Castanhinha et al., 2013; Cox & Angielczyk, 2014). The three recognized cryptodont families (Oudenodontidae, Rhachiocephalidae, and Geikiidae) were each recovered containing their ‘core’ taxa (i.e., *Oudenodon*, *Australobarbarus*, and *Tropidostoma* in Oudenodontidae; *Rhachiocephalus* and *Kitchinganomodon* in Rhachiocephalidae; and *Geikia*, *Aulacephalodon*, and *Pelanomodon* in Geikiidae). Unusually, however, *Odontocyclops* was recovered as an oudenodontid and *Syops* was recovered as a rhachiocephalid. Both of these results are novel: *Odontocyclops* was previously recovered as either the sister-taxon of *Idelesaurus* in a clade outside (Geikiidae + Rhachiocephalidae) (Kammerer, Angielczyk & Fröbisch, 2011; Kammerer, Angielczyk & Fröbisch, 2013; Castanhinha et al., 2013; Cox & Angielczyk, 2014) or as a geikiid (Boos et al., 2016) and *Syops* was previously recovered as either a dicynodontoid (Kammerer, Angielczyk & Fröbisch, 2011; Boos et al., 2016) or a geikiid (Castanhinha et al., 2013; Kammerer, Angielczyk & Fröbisch, 2013; Cox & Angielczyk, 2014). The position of *Odontocyclops* is based on five continuous characters (character 8, length of interpterygoid vacuity; character 12, ratio of length to height of mandibular fenestra; character 15, relative minimum and maximum widths of scapula; character 17, maximum width of distal end of radius; and character 18, ratio of posterior iliac process length to acetabular diameter) and two discrete state characters (character 52, state 1: small postparietal contribution to the skull roof, present in the other oudenodontids as well as *Kitchinganomodon*; and character 106, state 0: absence of ridge on edge of dentary symphysis, also coded for the other oudenodontids, *Syops*, *Rhachiocephalus*, *Kitchinganomodon*, and *Aulacephalodon*). The position of *Syops* is based on one continuous character (character 4: relative width of intertemporal bar) and two discrete state characters (character 46, state 2: parietal exposed in intertemporal bar as a thin crest, otherwise only coded for *Rhachiocephalus* and *Kitchinganomodon* among cryptodonts; and character 106, state 0: absence of ridge on edge of dentary symphysis).

*Bulbasaurus* was recovered as a geikiid in this analysis, sister-taxon to *Aulacephalodon*. The position of *Bulbasaurus* as a geikiid is supported by four discrete state characters (character 11, state 1: marked anterior expansion of preorbital region; character 34, state 1: transverse nasofrontal ridge; character 39, state 1: absence of postfrontal; and character 51, state 2: pineal foramen surrounded by raised edge). Its position as sister-taxon to *Aulacephalodon* is based solely on the absence of a postcaniniform crest (character 29, state 0). A postcaniniform crest on the posteroventral surface of the caniniform process is present in all other known cryptodonts. However, as discussed above, the postcaniniform crest is also present early in ontogeny in *Aulacephalodon* and lost when the tusks expand to full size, causing the caniniform process to bulge outwards. If the tusks did not develop in an *Aulacephalodon*-like animal, one would expect the postcaniniform crest to be retained. As such, this synapomorphy is questionable, considering the absence of tusks in *Pelanomodon* and *Geikia*. Indeed, it requires only a single additional step for *Bulbasaurus* to fall outside of Geikiinae (the clade containing *Aulacephalodon*, *Geikia*, and *Pelanomodon*) as a basal geikiid, a position better reflecting stratigraphy. Additional research on the patterns of character evolution in Cryptodontia is required to test this possibility. Cryptodonts in general suffer from a high degree of topological instability, low character support, and rampant homoplasy (as reflected in low consistency indices and extremely variable positions for taxa such as *Syops*), indicating that the current phylogenetic data set is not sufficient to robustly resolve their relationships.

## Conclusions

*Bulbasaurus phylloxyron* is identified as a new taxon of dicynodont, representing the earliest known record for the family Geikiidae and eliminating the ghost lineage for this clade in the *Tropidostoma* AZ. *Bulbasaurus* is most similar among known dicynodonts to its later relative *Aulacephalodon*, and exhibits many cranial characteristics of the latter taxon at substantially smaller skull size (13–16 cm dorsal skull length). The presence of ‘adult’ *Aulacephalodon* features in relatively small skulls suggests that these specimens represent mature individuals of *Bulbasaurus*, and that geikiids started out as relatively small animals. Similarity in size to *Tropidostoma* and absence of some of the more obvious features of later geikiids (e.g., broadened intertemporal region, protruding nasal bosses) previously obscured the true identity of *Bulbasaurus* specimens in collections. With the recognition of this taxon, however, Geikiidae can be added to the list of bidentalians (with Oudenodontidae and Rhachiocephalidae already represented) that first appear as rare faunal components of the *Tropidostoma* AZ. The continuing absence of dicynodontoids in this fauna remains problematic. Additional research attention should be given to revising existing collections of *Tropidostoma* AZ dicynodonts, as it is possible that early dicynodontoids could also be hiding among these specimens.

##  Supplemental Information

10.7717/peerj.2913/supp-1 Data S1 Phylogenetic data matrix

## Institutional abbreviations

BP: Evolutionary Studies Institute (formerly the Bernard Price Institute for Palaeontological Research), University of the Witwatersrand, Johannesburg, South Africa
BSPG: Bayerische Staatssammlung für Paläontologie und Geologie, Munich, Germany
CGP, CGS: Council for Geoscience, Pretoria, South Africa
NHMUK: The Natural History Museum, London, UK
SAM: Iziko Museums of South Africa, Cape Town, South Africa
TM: Ditsong, the National Museum of Natural History (formerly the Transvaal Museum), Pretoria, South Africa
USNM: National Museum of Natural History, Smithsonian Institution, Washington, D.C., USA.

## Additional Information and Declarations

### Data Availability

The following information was supplied regarding data availability: The raw data has been supplied as a Supplementary File.

### New Species Registration

The following information was supplied regarding the registration of a newly described species: *Bulbasaurus*: urn:lsid:zoobank.org:act:F21D10AF-277A-44D4-B374-BE89AF2B7541, *Bulbasaurus phylloxyron*: urn:lsid:zoobank.org:act:C80D31CA-86B1-43C4-9EA9-CED4A5852262, Publication LSID: urn:lsid:zoobank.org:pub:EB1EE1F8-C512-4272-BFA3-C8938E0806E5.

## References

[ref-1] Angielczyk KD (2002). Redescription, phylogenetic position, and stratigraphic significance of the dicynodont genus *Odontocyclops* (Synapsida: Anomodontia). Journal of Paleontology.

[ref-2] Angielczyk KD (2007). New specimens of the Tanzanian dicynodont *“Cryptocynodon” parringtoni* von Huene, 1942 (Therapsida, Anomodontia), with an expanded analysis of Permian dicynodont phylogeny. Journal of Vertebrate Paleontology.

[ref-3] Angielczyk KD, Fröbisch J, Smith RMH (2005). On the stratigraphic range of the dicynodont taxon *Emydops* (Therapsida: Anomodontia) in the Karoo Basin, South Africa. Palaeontologia Africana.

[ref-4] Angielczyk KD, Kurkin AA (2003). Phylogenetic analysis of Russian Permian dicynodonts (Therapsida: Anomodontia): implications for Permian biostratigraphy and Pangaean biogeography. Zoological Journal of the Linnean Society.

[ref-5] Angielczyk KD, Rubidge BS (2009). The Permian dicynodont *Colobodectes cluveri* (Therapsida, Anomodontia), with notes on its ontogeny and stratigraphic range in the Karoo Basin, South Africa. Journal of Vertebrate Paleontology.

[ref-6] Angielczyk KD, Rubidge BS (2013). Skeletal morphology, phylogenetic relationships and stratigraphic range of *Eosimops newtoni* Broom, 1921, a pylaecephalid dicynodonts (Therapsida, Anomodontia) from the Middle Permian of South Africa. Journal of Systematic Palaeontology.

[ref-7] Angielczyk KD, Rubidge BS, Day MO, Lin F (2016). A reevaluation of *Brachyprosopus broomi* and *Chelydontops altidentalis*, dicynodonts (Therapsida, Anomodontia) from the middle Permian *Tapinocephalus* Assemblage Zone of the Karoo Basin, South Africa. Journal of Vertebrate Paleontology.

[ref-8] Boonstra LD (1969). The fauna of the *Tapinocephalus* Zone (Beaufort beds of the Karoo). Annals of the South African Museum.

[ref-9] Boos ADS, Kammerer CF, Schultz CL, Soares MB, Ilha ALR (2016). A new dicynodont (Therapsida: Anomodontia) from the Permian of southern Brazil and its implications for bidentalian origins. PLOS ONE.

[ref-10] Botha J, Abdala F, Smith RMH (2007). The oldest cynodont: new clues on the origin and early diversification of the Cynodontia. Zoological Journal of the Linnean Society.

[ref-11] Botha J, Angielczyk KD (2007). An integrative approach to distinguishing the Late Permian dicynodont species *Oudenodon bainii* and *Tropidostoma microtrema* (Therapsida: Anomodontia). Palaeontology.

[ref-12] Botha-Brink J, Angielczyk KD (2010). Do extraordinarily high growth rates in Permo-Triassic dicynodonts (Therapsida, Anomodontia) explain their success before and after the end-Permian extinction?. Zoological Journal of the Linnean Society.

[ref-13] Brink AS (1986). Illustrated bibliographic catalogue of the Synapsida. Handbook of the South African geological survey, No. 10.

[ref-14] Broom R (1905). On the use of the term Anomodontia. Records of the Albany Museum.

[ref-15] Broom R (1935). On some new genera and species of Karroo fossil reptiles. Annals of the Transvaal Museum.

[ref-16] Broom R (1938). On two new anomodont genera. Annals of the Transvaal Museum.

[ref-17] Broom R (1940). On some new genera and species of fossil reptiles from the Karroo beds of Graaff-Reinet. Annals of the Transvaal Museum.

[ref-18] Broom R, Haughton SH (1917). Some new species of Anomodontia (Reptilia). Annals of the South African Museum.

[ref-19] Castanhinha R, Araújo R, Costa Júnior L, Angielczyk KD, Martins GG, Martins RMS, Chaouiya C, Beckmann F, Wilde F (2013). Bringing dicynodonts back to life: paleobiology and anatomy of a new emydopoid genus from the Upper Permian of Mozambique. PLOS ONE.

[ref-20] Cluver MA, Hotton III N (1981). The genera *Dicynodon* and *Diictodon* and their bearing on the classification of the Dicynodontia (Reptilia, Therapsida). Annals of the South African Museum.

[ref-21] Cox CB, Angielczyk KD (2014). A new endothiodont dicynodont (Therapsida, Anomodontia) from the Permian Ruhuhu Formation (Songea Group) of Tanzania and its feeding system. Journal of Vertebrate Paleontology.

[ref-22] Day MO, Güven S, Abdala A, Jirah S, Rubidge BS, Almond J (2015a). Youngest dinocephalian fossils extend the *Tapinocephalus* Zone, Karoo Basin, South Africa. South African Journal of Science.

[ref-23] Day MO, Ramezani J, Bowring SA, Sadler PM, Erwin DH, Abdala F, Rubidge BS (2015b). When and how did the terrestrial mid-Permian mass extinction occur? Evidence from the tetrapod record of the Karoo Basin, South Africa. Proceedings of the Royal Society B.

[ref-24] Fröbisch J (2007). The cranial anatomy of *Kombuisia frerensis* Hotton (Synapsida, Dicynodontia) and a new phylogeny of anomodont therapsids. Zoological Journal of the Linnean Society.

[ref-25] Fröbisch J (2013). Vertebrate diversity across the end-Permian extinction—separating biological and geological signals. Palaeogeography, Palaeoclimatology, Palaeoecology.

[ref-26] Fröbisch J, Reisz RR (2008). A new species of *Emydops* (Synapsida, Anomodontia) and a discussion of dental variability and pathology in dicynodonts. Journal of Vertebrate Paleontology.

[ref-27] Goloboff PA, Farris JS, Nixon KC (2008). TNT, a free program for phylogenetic analysis. Cladistics.

[ref-28] Irmis RB, Whiteside JH (2012). Delayed recovery of non-marine tetrapods after the end-Permian mass extinction tracks global carbon cycle. Proceedings of the Royal Society B.

[ref-29] Jasinoski SC, Cluver MA, Chinsamy A, Reddy BD, Kammerer CF, Angielczyk KD, Fröbisch J (2014). Anatomical plasticity in the snout of *Lystrosaurus*. Early evolutionary history of the Synapsida.

[ref-30] Kammerer CF (2011). Systematics of the Anteosauria (Therapsida: Dinocephalia). Journal of Systematic Palaeontology.

[ref-31] Kammerer CF (2016). A new taxon of cynodont from the *Tropidostoma* Assemblage Zone (upper Permian) of South Africa, and the early evolution of Cynodontia. Papers in Palaeontology.

[ref-32] Kammerer CF, Angielczyk KD (2009). A proposed higher taxonomy of anomodont therapsids. Zootaxa.

[ref-33] Kammerer CF, Angielczyk KD, Fröbisch J (2011). A comprehensive taxonomic revision of *Dicynodon* (Therapsida, Anomodontia) and its implications for dicynodont phylogeny, biogeography, and biostratigraphy. Society of Vertebrate Paleontology Memoir.

[ref-34] Kammerer CF, Angielczyk KD, Fröbisch J (2013). On the validity and phylogenetic position of *Eubrachiosaurus browni*, a kannemeyeriiform dicynodont (Anomodontia) from Triassic North America. PLOS ONE.

[ref-35] Kammerer CF, Angielczyk KD, Fröbisch J (2015a). Redescription of the geikiid *Pelanomodon* (Therapsida, Dicynodontia), with a reconsideration of ‘*Propelanomodon*’. Journal of Vertebrate Paleontology.

[ref-36] Kammerer CF, Angielczyk KD, Fröbisch J (2015b). Redescription of *Digalodon rubidgei*, an emydopoid dicynodont (Therapsida, Anomodontia) from the Late Permian of South Africa. Fossil Record.

[ref-37] Kammerer CF, Bandyopadhyay S, Ray S (2016). A new taxon of cistecephalid dicynodont from the upper Permian Kundaram Formation of India. Papers in Palaeontology.

[ref-38] Kammerer CF, Smith RMH, Day MO, Rubidge BS (2015). New information on the morphology and stratigraphic range of the mid-Permian gorgonopsian *Eriphostoma microdon* Broom, 1911. Papers in Palaeontology.

[ref-39] Keyser AW (1973). A re-evaluation of the genus *Tropidostoma* Seeley. Palaeontologia Africana.

[ref-40] King GM (1988). Anomodontia. Handbuch der Paläoherpetologie.

[ref-41] Kitching JW (1977). The distribution of the Karroo vertebrate fauna. Bernard Price Institute for Palaeontological Research Memoir.

[ref-42] Kurkin AA (2000). New dicynodonts from the Upper Permian of the Vyatka Basin. Paleontological Journal.

[ref-43] Modesto SP, Rubidge BS, Welman J (2002). A new dicynodont therapsid from the lowermost Beaufort Group, Upper Permian of South Africa. Canadian Journal of Earth Sciences.

[ref-44] Newton ET (1893). On some new reptiles from the Elgin Sandstone. Philosophical Transactions of the Royal Society of London B.

[ref-45] Nopcsa F (1923). Die familien der reptilien.

[ref-46] Osborn HF (1903). On the primary division of the Reptilia into two sub-classes, Synapsida and Diapsida. Science.

[ref-47] Owen R (1860). On the orders of fossil and recent Reptilia, and their distribution in time. Report of the Twenty-Ninth Meeting of the British Association for the Advancement of Science.

[ref-48] Ray S (2005). *Lystrosaurus* (Therapsida, Dicynodontia) from India: taxonomy, relative growth and cranial dimorphism. Journal of Systematic Palaeontology.

[ref-49] Retallack GJ, Smith RMH, Ward PD (2003). Vertebrate extinction across Permo-Triassic boundary in Karoo Basin, South Africa. Geological Society of America Bulletin.

[ref-50] Réy K, Amiot R, Fourel F, Rigaudier T, Abdala F, Day MO, Fernandez V, Fluteau F, France-Lanord C, Rubidge BS, Smith RMH, Viglietti P, Zipfel B, Lécuyer C (2016). Global climate perturbations during the Permo-Triassic extinctions recorded by continental tetrapods from South Africa. Gondwana Research.

[ref-51] Rubidge BS (1995). Biostratigraphy of the Beaufort Group (Karoo Supergroup).

[ref-52] Rubidge BS, Day MO, Barbolini N, Hancox PJ, Choiniere JN, Bamford MK, Viglietti PA, McPhee BW, Jirah S, Linol B, De Wit MJ (2016). Advances in nonmarine Karoo biostratigraphy: significance for understanding basin development. Origin and evolution of the Cape Mountains and Karoo Basin.

[ref-53] Rubidge BS, Erwin DH, Ramezani J, Bowring SA, De Klerk WJ (2013). High-precision temporal calibration of Late Permian vertebrate biostratigraphy: U-Pb zircon constraints from the Karoo Supergroup, South Africa. Geology.

[ref-54] Seeley HG (1889). Researches on the structure, organization, and classification of the fossil Reptilia.—VI. On the Anomodont Reptilia and their allies. Philosophical Transactions of the Royal Society of London B.

[ref-55] Seeley HG (1898). On *Oudenodon* (*Aulacocephalus*) *pithecops* from the *Dicynodon* Beds of East London, Cape Colony. Geological Magazine.

[ref-56] Sidor CA, Hopson JA, Keyser AW (2004). A new burnetiamorph therapsid from the Teekloof Formation, Permian, of South Africa. Journal of Vertebrate Paleontology.

[ref-57] Sidor CA, Smith RMH (2007). A second burnetiamorph therapsid from the Permian Teekloof Formation of South Africa and its associated fauna. Journal of Vertebrate Paleontology.

[ref-58] Smith RMH (1987). Morphology and depositional history of exhumed Permian point-bars in the southwestern Karoo, South Africa. Journal of Sedimentary Petrology.

[ref-59] Smith RMH (1993). Vertebrate taphonomy of Late Permian floodplain deposits in the southwestern Karoo Basin, South Africa. PALAIOS.

[ref-60] Smith RMH, Botha-Brink J (2014). Anatomy of a mass extinction: sedimentological and taphonomic evidence for drought-induced die-offs at the Permo-Triassic boundary in the marin Karoo Basin, South Africa. Palaeogeography, Palaeoclimatology, Palaeoecology.

[ref-61] Smith RMH, Rubidge BS, van der Walt M, Chinsamy A (2012). Therapsid biodiversity patterns and environments of the Karoo Basin, South Africa. *Forerunners of mammals: radiation, histology, biology*.

[ref-62] Smith RMH, Ward PD (2001). Pattern of vertebrate extinctions across an event bed at the Permian-Triassic boundary in the Karoo Basin of South Africa. Geological Survey of America Bulletin.

[ref-63] Sullivan CR, Reisz RR, Smith RMH (2003). The Permian mammal-like herbivore *Diictodon*, the oldest known example of sexually dimorphic armament. Proceedings of the Royal Society of London B Biological Sciences.

[ref-64] Toerien MJ (1955). Important new Anomodontia. Palaeontologia Africana.

[ref-65] Tollman SM, Grine FE, Hahn BD (1980). Ontogeny and sexual dimorphism in *Aulacephalodon* (Reptilia, Anomodontia). Annals of the South African Museum.

[ref-66] Viglietti PA, Smith RMH, Angielczyk KD, Kammerer CF, Fröbisch J, Rubidge BS (2016). The *Daptocephalus* Assemblage Zone (Lopingian), South Africa: a proposed biostratigraphy based on a new compilation of stratigraphic ranges. Journal of African Earth Sciences.

[ref-67] Ward PD, Botha J, Buick R, De Kock MO, Erwin DH, Garrison G, Kirschvink J, Smith RMH (2005). Abrupt and gradual extinction among Late Permian land vertebrates in the Karoo Basin, South Africa. Science.

[ref-68] Wyllie A (2003). A review of Robert Broom’s therapsid holotypes: have they survived the test of time?. Palaeontologia Africana.

